# HAG1 and SWI3A/B control of male germ line development in *P. patens* suggests conservation of epigenetic reproductive control across land plants

**DOI:** 10.1007/s00497-021-00409-0

**Published:** 2021-04-11

**Authors:** Anne C. Genau, Zhanghai Li, Karen S. Renzaglia, Noe Fernandez Pozo, Fabien Nogué, Fabian B. Haas, Per K. I. Wilhelmsson, Kristian K. Ullrich, Mona Schreiber, Rabea Meyberg, Christopher Grosche, Stefan A. Rensing

**Affiliations:** 1 Plant Cell Biology, Department of Biology, University of Marburg, Marburg, Germany; 2 Department of Plant Biology, Southern Illinois University, Carbondale, IL 62901 USA; 3 Institut Jean-Pierre Bourgin, INRAE, Université Paris-Saclay, 78000 Versailles, AgroParisTech France; 4 Present Address: Department of Evolutionary Genetics, Max Planck Institute for Evolutionary Biology, Plön, Germany; 5 BIOSS Centre for Biological Signaling Studies, University of Freiburg, Freiburg, Germany; 6 LOEWE Center for Synthetic Microbiology (SYNMIKRO), Philipps University of Marburg, Marburg, Germany

**Keywords:** *Physcomitrium*, *Marchantia*, Spermatozoid, Gametangia, Fertilization, Germ line

## Abstract

**Key message:**

Bryophytes as models to study the male germ line: loss-of-function mutants of epigenetic regulators HAG1 and SWI3a/b demonstrate conserved function in sexual reproduction.

**Abstract:**

With the water-to-land transition, land plants evolved a peculiar haplodiplontic life cycle in which both the haploid gametophyte and the diploid sporophyte are multicellular. The switch between these phases was coined alternation of generations. Several key regulators that control the bauplan of either generation are already known. Analyses of such regulators in flowering plants are difficult due to the highly reduced gametophytic generation, and the fact that loss of function of such genes often is embryo lethal in homozygous plants. Here we set out to determine gene function and conservation via studies in bryophytes. Bryophytes are sister to vascular plants and hence allow evolutionary inferences. Moreover, embryo lethal mutants can be grown and vegetatively propagated due to the dominance of the bryophyte gametophytic generation. We determined candidates by selecting single copy orthologs that are involved in transcriptional control, and of which flowering plant mutants show defects during sexual reproduction, with a focus on the under-studied male germ line. We selected two orthologs, *SWI3a/b* and *HAG1*, and analyzed loss-of-function mutants in the moss *P. patens*. In both mutants, due to lack of fertile spermatozoids, fertilization and hence the switch to the diploid generation do not occur. Pp*hag1* additionally shows arrested male and impaired female gametangia development. We analyzed *HAG1* in the dioecious liverwort *M. polymorpha* and found that in Mp*hag1* the development of gametangiophores is impaired. Taken together, we find that involvement of both regulators in sexual reproduction is conserved since the earliest divergence of land plants.

**Supplementary Information:**

The online version contains supplementary material available at 10.1007/s00497-021-00409-0.

## Introduction

All land plants (Embryophyta) possess a haplodiplontic life cycle, i.e., both the haploid and diploid generation (phase) divide mitotically and are hence multicellular. In the process of sexual reproduction the haploid gametophyte develops sperm and egg cells, while the diploid sporophyte undergoes meiosis, yielding spores from which in turn the gametophyte develops. This “alternation of generations” (Hofmeister (1979) is in contrast to e.g., diplontic multicellular animals (Metazoa) in which only the diploid phase is multicellular, or haplontic streptophytic algae (sister lineage to land plants), in which only the haploid phase is multicellular. Despite these differences, eukaryotic life cycles are united by the diploid zygote arising from the fusion of two haploid gametes. Intriguingly, the haploid-to-diploid transition is an ancestral eukaryotic feature that is controlled by homeodomain (HD) transcription factors (TF) of the TALE (three amino acid loop extension) class (Joo et al. 2018).

In most eukaryotic lineages the male gametes bear flagella and are motile. A prerequisite for successful alternation of generations is the male gamete reaching the female gamete. This is enacted, e.g., by pollen in flowering plants and conifers, while most other plant lineages rely on flagellated male gametes (Renzaglia and Garbary 2001). Flagellated sperm cells are an ancestral character of eukaryotes (Stewart and Mattox (1975), Mitchell 2007) that has been secondarily lost in the Zygnematales (belonging to the streptophytic algae), the conifers and Gnetales, and the flowering plants. In well-studied flowering plant models such as *Arabidopsis thaliana* the male gametophyte has been secondarily reduced, characterized by a loss of flagella and mitosis occurring inside the microscopic pollen. To further our understanding of how the plant male germ line is controlled we examined genes putatively involved in this process, which is directly linked to sexual reproduction.

The HD-TALE TFs KNOX/BELL have been shown to control gamete fusion, which is mediated through the flagella, in the unicellular alga *Chlamydomonas reinhardtii* (Lee et al. 2008). The authors’ hypothesis that BELL orthologs might control similar processes in land plants has recently been demonstrated in the moss *Physcomitrium patens* (previously *Physcomitrella patens*) (Rensing et al. 2020), where members of this family are involved in proper zygote formation (Horst et al. 2016a; Ortiz-Ramirez et al. 2017) and are implicated in control of male fecundity (Meyberg et al. 2020). Evolutionary-developmental studies in the moss have also revealed that apogamy, the formation of the diploid body plan from haploid tissue, is prevented epigenetically via the polycomb repressive complex 2, PRC2 (Mosquna et al. 2009; Okano et al. 2009). Also, the formation of the haploid body plan from diploid cells, termed apospory, is controlled by KNOX2 TFs (Sakakibara et al. 2013), and LEAFY controls the first division of the zygote (Tanahashi et al. 2005). More recently, studies in the liverwort model *Marchantia polymorpha* revealed that the bHLH TF BNB controls the protrusion of gametangiophores, while the flowering plant ortholog controls generative cell specification in pollen (Hisanaga et al. (2019) (cf. Fig. 14). Also, RWP-RK TFs that control flowering plant egg cell differentiation control the differentiation of antheridial cells into spermatid mother cells and egg cell differentiation in *M. polymorpha* (MpRKD) (Rovekamp et al. 2016; Koi et al. 2016). MYB TFs that are responsible for flowering plant sperm cell formation control the last step of spermatozoid formation in the liverwort (MpDUO1) (Hisanaga et al. 2019). These examples demonstrate the power of bryophyte models for studying sexual reproduction: (i) the sexual and asexual generations are easily tractable, (ii) loss-of-function mutants that are embryo lethal in homozygous flowering plants can usually be grown and propagated vegetatively and (iii) the development of gametangia can be considered equivalent to flower development (see Rensing 2016) for commonalities of moss and flowering plant life cycles. During land plant evolution, gametophytic genes were frequently co-opted for sporophyte developmental processes; hence, structures fulfilling the same function (e.g., sexual reproduction or photosynthesis) in the two alternating generations are often controlled by the same genes (Kenrick 2017,2018).

Gene duplication and subsequent sub- and neofunctionalization drive plant morphogenetic evolution (Rensing 2014). The gene balance hypothesis (Birchler and Veitia 2012) states that genes affected by dosage sensitivity (such as heterodimeric transcription factors, TFs) tend to be retained after polyploidization events, and indeed, there is evidence that the number of TFs increases with plant complexity and polyploidization (Lang et al. 2010; Peer et al. 2009). Dosage-sensitive genes require the proper stoichiometry of their gene products for correct function, e.g., for protein–protein interaction. After a whole genome duplication (WGD) the dosage of all genes is increased, while small-scale duplications may lead to stoichiometric imbalance of the respective complexes (Edger and Pires 2009). There are TF genes that are usually single copy in most plant genomes—a well-studied example being the floral regulator LEAFY that potentially evolves via “promiscuous” intermediates of its DNA binding domain (Sayou et al. 2014a). Apparently, copies of LEAFY are usually not retained, a situation explained by the Selected Single Copy Gene Hypothesis: it amends the gene balance hypothesis by stating that duplicate retention of such genes is selected against, because the gene products would be under dosage imbalance (Edger and Pires 2009; Duarte et al. 2010).

As shown in the examples outlined above, sexual reproduction as well as male and female germ line development is controlled both by TFs acting on DNA in sequence-specific fashion and by transcriptional regulators (TRs) that act via epigenetic regulation or on protein–protein interaction level. Often, such key regulators are encoded by singly copy genes (Mosquna et al. 2009; Okano et al. 2009; Sayou et al. 2014b). We considered such genes prime candidates to evolve under the Selected Single Copy Gene Hypothesis. Another example for dosage-sensitive genes is ribosomal complexes: the duplicates of nuclear ribosomal protein genes are usually retained after a WGD (Papp et al. 2003; Aury et al. 2006). In contrast, following the Selected Single Copy Hypothesis, the duplication of the ribosomal protein RPS13, which encodes a subunit of the mitochondrial organellar ribosomal complex, might lead to dosage effects, resulting in counter-selection against retention of the duplicate (Edger and Pires 2009).

In order to further elucidate the conservation of plant sexual reproduction, we performed a phylogenetic candidate gene screening to identify unstudied bryophyte TF/TR genes potentially involved in controlling the male germ line and hence sexual reproduction. To do so, we employed an orthology detection tool (proteinortho) (Lechner et al. 2011), the protein-family annotation tool TAPscan (Lang et al. 2010; Wilhelmsson et al. 2017), literature search and phylogenetic inference.

Using this approach, we identified two promising candidates and studied their role in the control of bryophyte sexual reproduction. Our data show that both are master regulators, the loss of which leads to a severe phenotype based primarily on defects in the male germ line.

## Results and discussion

### Phylogenetic screening for conserved single copy TF/TR

As outlined in Introduction, we considered single copy orthologs as prime candidates to control important stages in the plant life cycle. Furthermore, we expected TF/TR as prime candidates to have evolved under the Selected Single Copy Gene Hypothesis, i.e., to be present as singly copy orthologs. Indeed, single copy ortholog clusters (cf. Fig. S1 and Methods) contain twice as many TF/TR genes as expected by chance, which is a significant enrichment (*p* = 4E−07, Chi squared test). Single copy genes are expected to be typically represented by a family size of 1 in a comparison across species. Using TAPscan (Wilhelmsson et al. 2017), we determined the mode (i.e., the value that appears most often) of TF/TR gene families in 58 plant genomes. We found that among those families with a mode of 1 we could indeed detect (among others) LEAFY and PcG FIE, for which the involvement in the alternation of generations is well established (Mosquna et al. 2009; Lohmann et al. 2001), supporting our claim.

Our main focus was *A. thaliana* as well as the three bryophytes *Physcomitrium patens*, *Marchantia polymorpha* and *Anthoceros agrestis*, the former because *A. thaliana* is the best characterized plant species in terms of mutant phenotypes, the latter three as representatives of all three lineages of bryophytes (mosses, liverworts and hornworts). Using proteinortho (Lechner et al. 2011) we identified ortholog clades comprising single copy genes for bryophytes as well as *A. thaliana*. Filtering for TF/TR (Wilhelmsson et al. 2017), as potentially important regulatory hubs, resulted in determination of 23 clades (Fig. S1). For those we confirmed orthology status via phylogenetic analyses and performed literature searches (looking primarily into loss-of-function in *A. thaliana*) as well as expression profile screening during sexual reproduction (for details see Table S1). We selected two genes based on their *A. thaliana* loss-of-function phenotype (focusing on control of the male germ line), their expression during sexual reproduction, and the fact that they had not previously been studied in bryophytes.

### HAG1

Histone acetylation is a potential regulatory mechanism involved in transcriptional activation (Burk et al. 2003) and therefore associated with many developmental and biological processes in eukaryotes (Verdone et al. 2005). Histone acetyltransferases are highly conserved in all eukaryotic lineages (Kim et al. 2015; Aquea et al. 2017). The *A. thaliana HAG1* ortholog AT3G54610 (Kim et al. 2015), also known as *GCN5*, was shown to be predominantly involved in the acetylation of H3K14 (Benhamed et al. 2006; Earley et al. 2007; Mahrez et al. 2016). As a fundamental regulator, HAG1 is associated with a high number of developmental processes (Aquea et al. 2017), e.g., timing of juvenile-to-adult phase transition (Kim et al. 2015), inflorescence meristem and stamen development (Cohen et al. 2009), and miRNA production (Kim et al. 2009). Mutations of *HAG1* were found to be associated with the alternation of generations, namely abnormal flower development and reduced fertility (Bertrand et al. 2003; Vlachonasios et al. 2003). Observed loss of fecundity was attributed to a reduced stamen height in early-arising flowers and an increase in stamen number in early-arising flowers in contrast to late flowers that are able to produce small siliques with a few seeds (Vlachonasios et al. 2003).

**Fig. 1:**
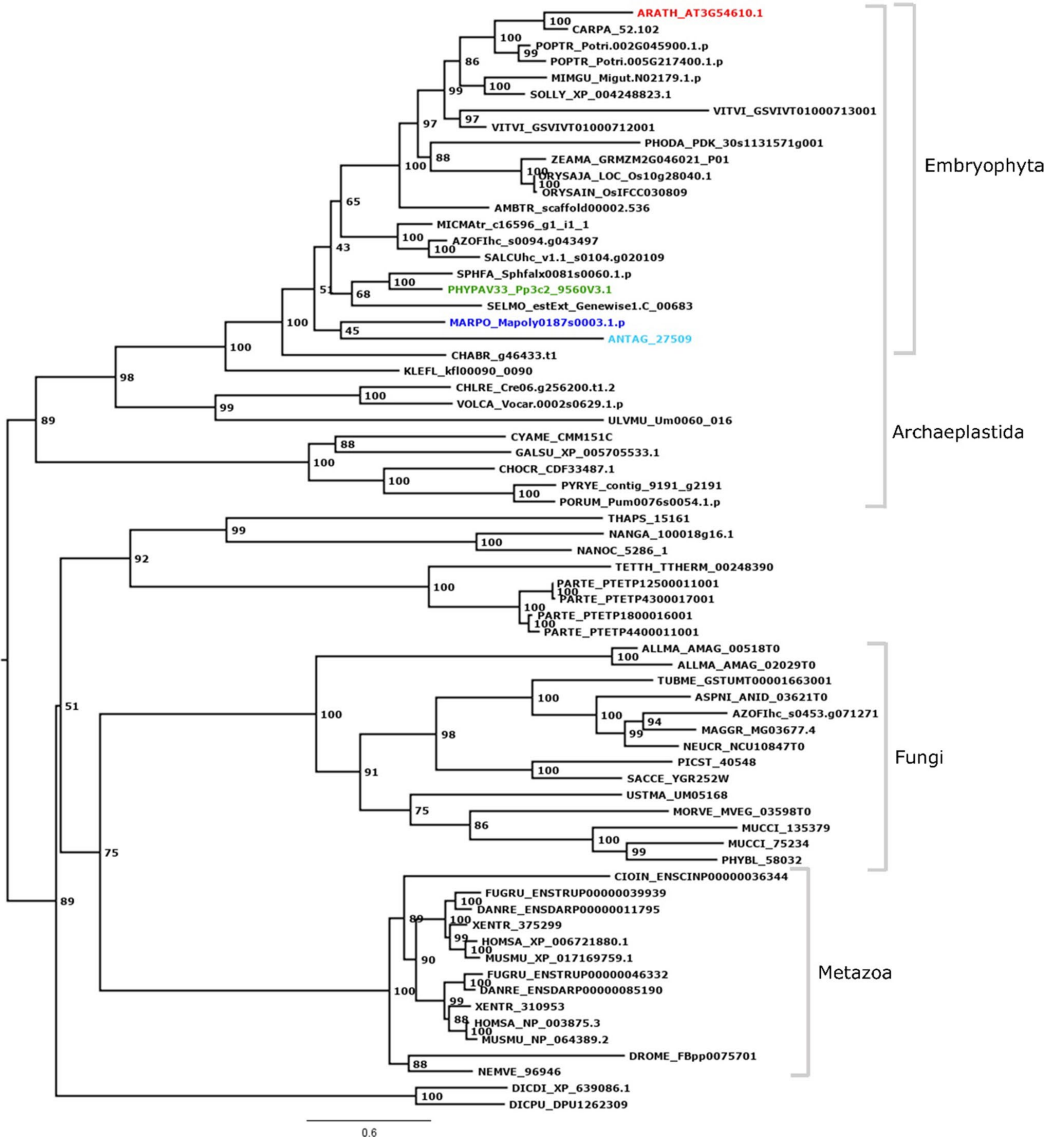
HAG1 phylogenetic analysis. Midpoint-rooted maximum likelihood tree of the HAG1 family across selected eukaryotes. There are clear single copy orthologs of the *A. thaliana* gene (red) in the three bryophyte species of interest, *P. patens* (moss, green), *A. agrestis* (hornwort, turquoise) and *M. polymorpha* (liverwort, blue). Support values at the nodes represent bootstrap support. Species names are abbreviated in a five letter code where the first three letters represent the species and the latter two the genus, e.g., *ORYza SAtiva* = ORYSA [red algae: CYAME = *Cyanidioschyzon merolae*, GALSU = *Galdieria sulphuraria*, CHOCR = *Chondrus crispus*, PORUM = *Porphyra umbilicalis*, PYRYE = *Pyropia yezoensis*; chlorophyte algae: CHLRE = *Chlamydomonas reinhardtii*, ULVMU = *Ulva mutabilis*, VOLCA = *Volvox carteri*; streptophyte algae: KLEFL = *Klebsormidium nitens*, CHABR = *Chara braunii*; bryophytes: ANTAG = *Anthoceros agrestis*, MARPO = *Marchantia polymorpha*, PHYPA = *Physcomitrium patens*, SPHFA = *Sphagnum fallax*; SELMO = *Selaginella moellendorffii* (lycophyte); ferns: AZOFI = *Azolla filiculoides*, SALCU = *Salvinia cucullate*, MICMA = *Microlepia marginata*; angiosperms: monocots, ZEAMA = *Zea mays*, ORYSA = *Oryza sativa*, PHODA = *Phoenix dactylifera;* dicots, ARATH = *Arabidopsis thaliana*, POPTR = *Populus trichocarpa*, CARPA = *Carica papaya*, SOLLY = *Solanum lycopersicum*, MIMGU = *Mimulus guttatus*, VITVI = *Vitis vinifera*, PHODA = *Phoenix dactylifera*; ANA grade: AMBTR = *Amborella trichopoda;* fungi: ALLMA = *Allomyces macrogynus*, MORVE = *Mortierella verticillata*, MUCCI = *Mucor circinelloides*, PHYBL = *Phycomyces blakesleeanus*, USTMA = *Ustilago maydis*, SACCE = *Saccharomyces cerevisiae*, PICST = *Scheffersomyces stipitis*, TUBME = *Tuber melanosporum*, ASPNI = *Aspergillus nidulans*, NEUCR = *Neurospora crassa*, MAGGR = *Magnaporthe grisea*; amoebozoa: DICPU = *Dictyostelium purpureum*, DICDI = *Dictyostelium discoideum*; SAR: THAPS = *Thalassiosira pseudonana*, NANOC = *Nannochloropsis oceanica* CCMP1779, NANGA = *Nannochloropsis gaditana*, TETTH = *Tetrahymena thermophila*, PARTE = *Paramecium tetraurelia*; invertebrates: CIOIN = *Ciona intestinalis*, NEMVE = *Nematostella vectensis*, DROME = *Drosophila melanogaster*; vertebrates: HOMSA = *Homo sapiens*, FUGRU = *Fugu rubripes*, XENTR = *Xenopus tropicalis*, MUSMU = *Mus musculus*, DANRE = *Danio rerio*]

HAG1/GCN5 belongs to the GNAT TR family defined by the presence of the GNAT acetyltransferase domain (PFAM PF00583) (Wilhelmsson et al. 2017). Multiple sequence alignment shows the conservation of the GNAT domain with its Coenzyme A binding pocket (Fig. S2). Our phylogenetic analysis of the HAG1 clade (Fig. 1) shows that there are single copy orthologs of the single *A. thaliana* gene (red) in the moss *P. patens* (green), the hornwort *A. agrestis* (turquoise) and the liverwort *M. polymorpha* (blue). The phylogenetic analysis suggests presence of *HAG1* in the last eukaryotic common ancestor (LECA) and shows that in most eukaryotic lineages it is predominantly a single copy gene. As an exception, the gene was apparently duplicated in the most recent common ancestor of vertebrates, and there are recently acquired paralogs for example in poplar and grapevine. Interestingly, the analyzed gymnosperm species lost *HAG1*.

### SWI3A/B

Chromatin-remodeling complexes (CRC) constitute a switch point between signaling pathways and chromatin-based control of transcription. They are involved in transcriptional activation as well as repression (Sarnowski et al. 2005; Roberts and Orkin 2004). SWI3 is part of the SWITCH/SUCROSE NONFERMENTING (SWI/SNF) CRC, which is involved in ATP-dependent alteration of DNA-histone contacts (Sarnowski et al. 2005). According to the TAPscan classification (Wilhelmsson et al. 2017) SWI3 is part of the MYB-related TF family characterized by the Myb_DNA-binding domain (PF00249). However, according to the PFAM domain description the domain family contains not only DNA binding domains but also the SANT domain (Aasland et al. 1996). The two domains show high structural similarity (Grune et al. 2003), but the SANT domain is predicted to be incompatible with DNA binding. Multiple sequence alignment shows the conservation of the SANT/MYB domain with the MYB domains’ potentially DNA-binding residues (Fig. S3). Based on the conflicting evidence, SWI3 cannot clearly be classified as TF or TR.

**Fig. 2:**
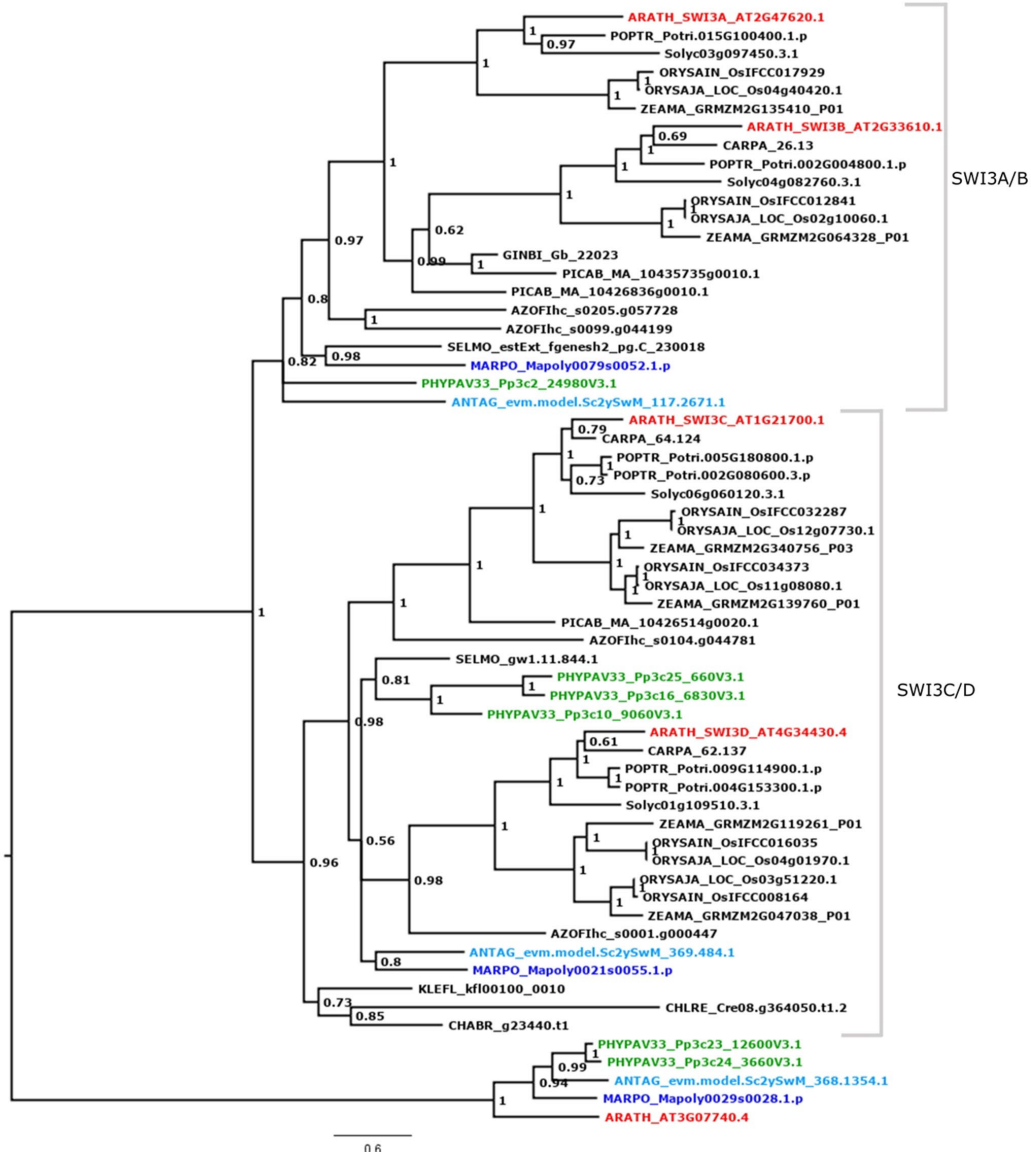
SWI3 phylogenetic analysis. Midpoint-rooted Bayesian phylogenetic tree of plant SWI3 proteins. The phylogenetic analysis distinguishes the four *A. thaliana* SWI-like proteins (A, B, C, D; red). The SWI3A/B clade shows single copy orthologs for the three bryophyte species of interest, P. patens (moss, green), A. agrestis (hornwort, turquoise) and *M. polymorpha* (liverwort, blue). Support values at the nodes represent posterior probabilities of Bayesian inference, and the outgroup represents the longest internal branch. Species names are abbreviated in a five letter code where the first three letters represent the species and the latter two the genus, e.g., *ORYza SAtiva*, ORYSA (rice) [angiosperms: dicots, ARATH = *A. thaliana* (thale cress), CARPA = *Carica papaya* (papaya), POPTR = *Populus trichocarpa* (poplar), SOLLY = *Solanum lycopersicum* (tomato); monocots: ORYSAIN = *Oryza sativa indica* (rice), ORYSAJA = *Oryza sativa japonica* (rice), ZEAMA = *Zea mays* (corn); gymnosperms: PICAB = *Picea abies* (Norway spruce), GINBI = *Ginkgo biloba*; AZOFI = *Azolla filiculoides* (water fern); SELMO = *Selaginella moellendorffii* (lycophyte); bryophytes: PHYPA = *Physcomitrium patens* (moss), MARPO = *Marchantia polymorpha* (liverwort), ANTAG = *Anthoceros agrestis* (hornwort); streptophyte algae: CHABR = *Chara braunii*, KLEFL = *Klebsormidium flaccidum*; CHLRE = *Chlamydomonas reinhardtii* (chlorophyte alga)]

The genome of *A. thaliana* encodes four SWI3-like proteins, which are diverse in their function. The At*swi3a* and At*swi3b* mutants have severe defects in embryo development, causing a lethal arrest at the early globular stage (Sarnowski et al. 2005). The phylogenetic analysis of SWI3 (Fig. 2) shows that there are four clades of SWI3-like proteins in *A. thaliana*, with SWI3A/B and SWI3C/D clustering in distinct subtrees. The SWIC/D clade probably represents the ancestral clade, on account of algal sequences clustering exclusively in this clade. Within the SWI3C/D clade there are no clear orthologs for either SWI3C or SWI3D in the bryophytes studied. Mutations in *AtSWI3C* lead to a semi-dwarfed body plan, root elongation is inhibited, the leaves show a curly morphogenesis, stamen development is abnormal, and fecundity is impaired. Mutations in *AtSWI3D* cause dwarfism, abnormal flower organ development and male and female sterility (Sarnowski et al. 2005).

Similar to C/D, the bryophyte sequences of the SWI3A/B clade are sister to the combined A and B clades of the angiosperms. Most probably the duplications yielding A/B and C/D occurred during seed plant evolution. There are single copy orthologs to the seed plant SWI3A/B clade in the moss *P. patens* (green), the hornwort *A. agrestis* (turquoise) and the liverwort *M. polymorpha* (blue).

### Expression, localization and loss-of-function mutants in *P. patens*

Both candidate genes show increased expression during sexual reproduction (Fig. S4), in particular in the male germ line. Since they are TF/TR, the gene products are expected to be localized in the nucleo-/cytoplasm. This localization was confirmed in vivo via C-terminal in frame GFP tagging (Fig. S5). To determine the gene function of the two candidate genes in bryophytes, loss-of-function mutants were obtained with the help of CRISPR-Cas9 gene perturbation (Figs. S6, S7). Mutants were genotyped by PCR and sequencing of amplified PCR products. Mutants were detected by a shortened (approximately 200 bp) PCR product. The deletion led to either a shortened protein, if the deletion was in-frame, or a frameshift resulting in a newly created stop codon (Fig. S7). For every candidate gene at least five independent mutant lines were identified. Three independent mutant lines and the control (accession “Reute”) were used for further analysis of sporophyte development as well as crossing analysis. Detailed morphological analyses as well as RNA-seq analysis were carried out for one mutant line each as well as the control.

### Sporophyte development is impaired in both mutants due to early arrest at the gametangial stage

**Fig. 3:**
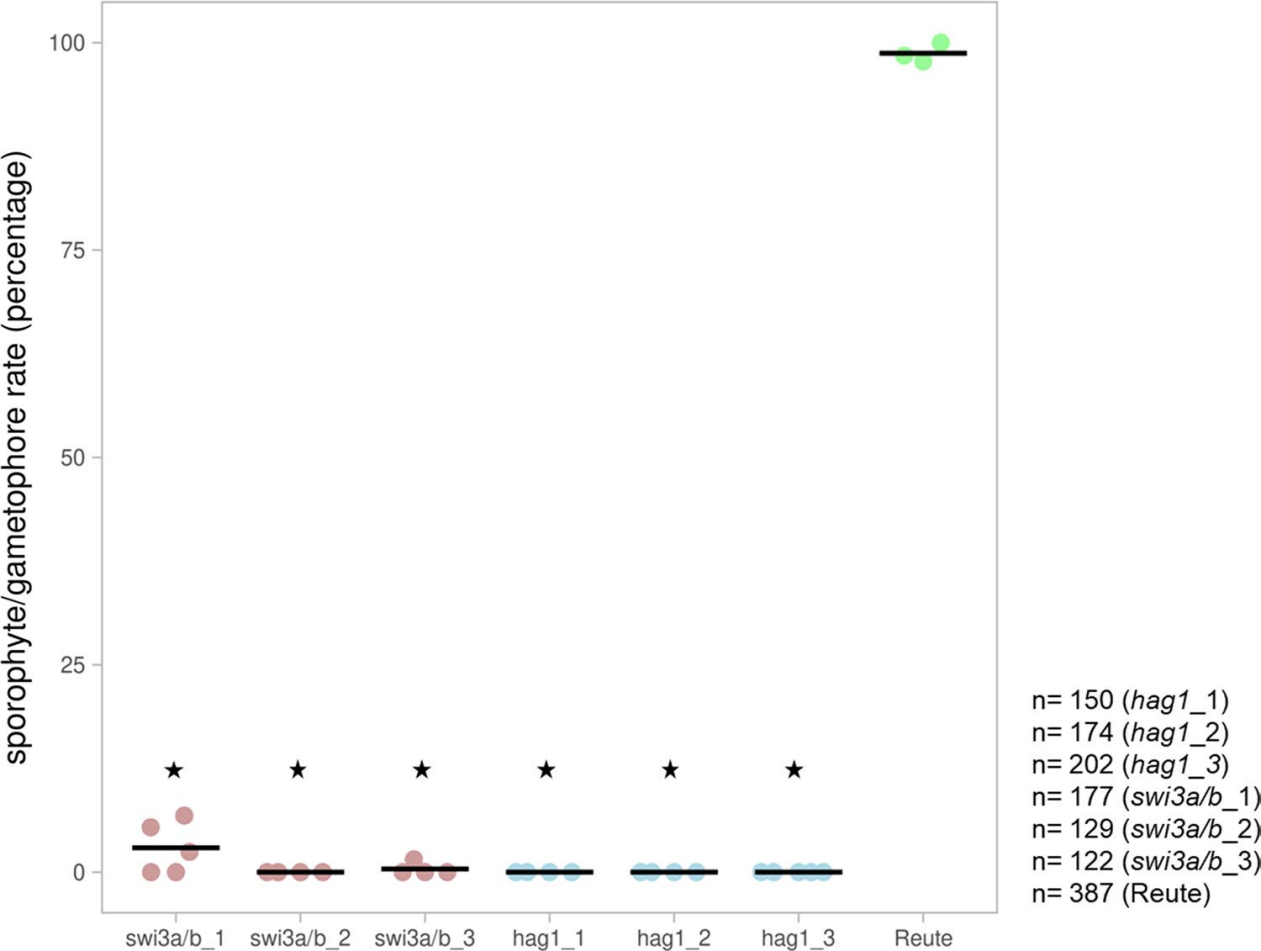
Sporophytes per gametophore. The control (Reute, green) shows the expected selfing rate/number of sporophytes per gametophore of in average 99%. Pp*swi3a/b* (red) and Pp*hag1* (blue) show significantly less sporophytes per gametophore (*p* < 0.01, Fisher’s exact test, asterisks). At least three independent replicates (dots) were performed for the mutant lines as well as the control; the total number of gametophores analyzed per mutant/control is shown to the right. The number of sporophytes per gametophore was calculated as percentage relative to the total number of gametophores. Averages of replicates are shown as horizontal lines

**Fig. 4:**
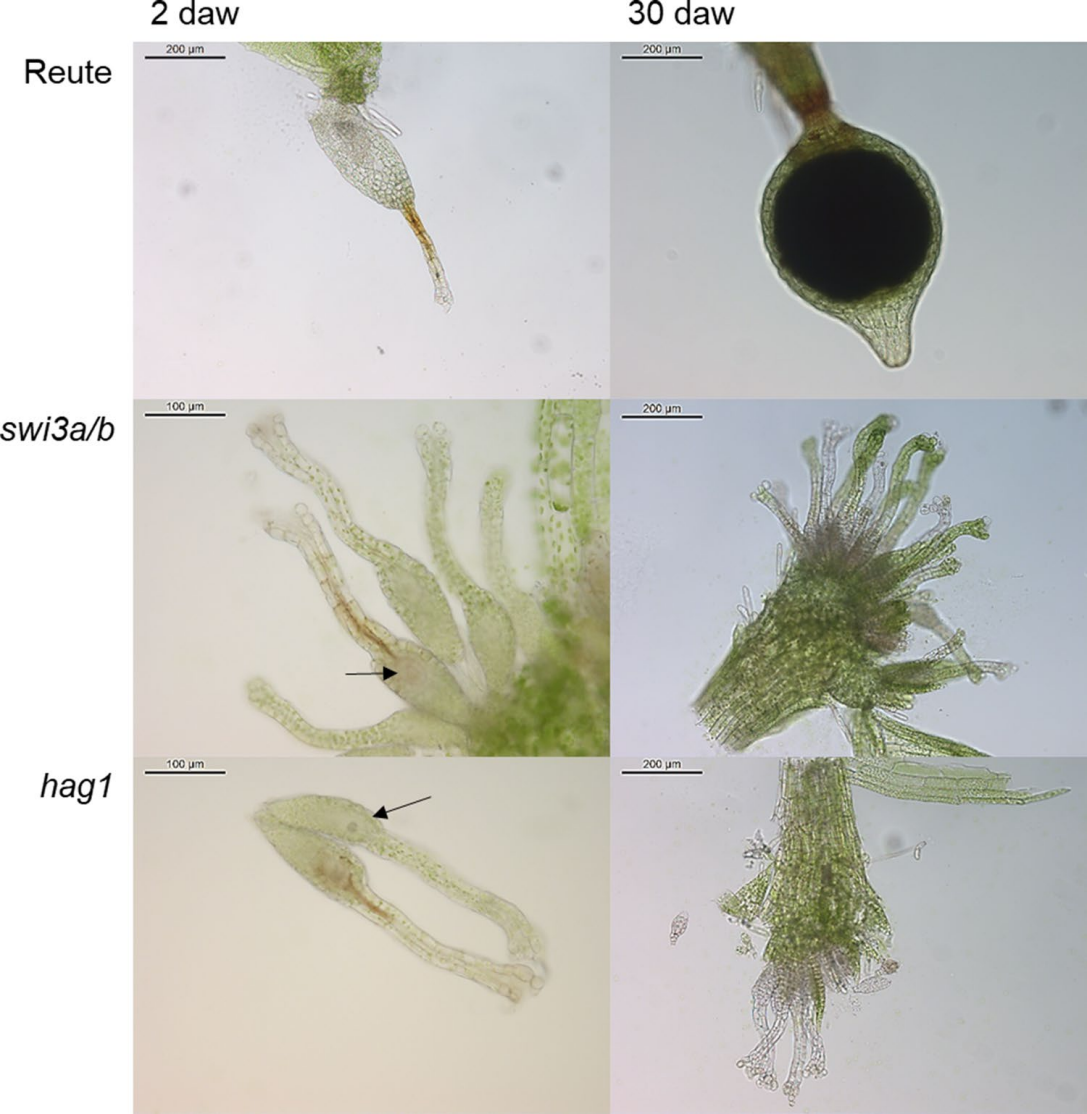
Phenotypic analysis of *swi3a/b* and *hag1* compared to control after watering. Two days after watering, Reute (control) developed early stage (ES) sporophytes, recognizable by the swollen archegonial cavity, while the mutants were arrested at the archegonial stage, with egg cells still being visible inside the cavity (arrows). Reute developed mature brown (B) sporophytes 30 days after watering, while both mutants developed an abundance of archegonia. Developmental stages according to Hiss et al. (2017). Note the brown coloration of archegonial neck cells 2daw, that has been described to occur in the wildtype upon fertilization, but apparently occurs in the mutants despite lack of fertilization

To check for impairment of sexual reproduction, juvenile (asexual) gametophores were placed under inductive conditions for development of gametangia and subsequently sporophytes. Under these conditions, Reute control plants show on average 99% of sporophytes per leafy shoot (gametophore). In contrast, all lines of both deletion mutants show a significant reduction of sporophytes per gametophore (Fig. 3). For the subsequent detailed phenotypical analyses, *swi3a/b_2* and *hag_1* were selected (cf. Fig. S7, Table S2). The control (Reute background) undergoes normal development leading to an early sporophyte (ES) stage and a brown mature sporophyte (B) two and 30 days following fertilization, respectively (Hiss et al. 2017). Two days after watering (daw) control and mutants bear archegonia with a brown coloration of the archegonial neck canal cells (Fig. 4). Such brown coloration is usually observed upon entry of spermatozoids into the archegonium (Tanahashi et al. 2005), but occurs in the mutants although no embryo is formed. If no fertilization occurs, *P. patens* develops superfluous gametangia (Landberg et al. 2013; Sanchez-Vera et al. 2017). Indeed, both mutants developed cauliflower-like bundles of gametangia 30 days after fertilization (Fig. 4). A similar phenotype recently was described for another fertilization-impaired mutant (Meyberg et al. 2020).

### Crossing analyses demonstrate predominantly male germ line impairment

**Fig. 5:**
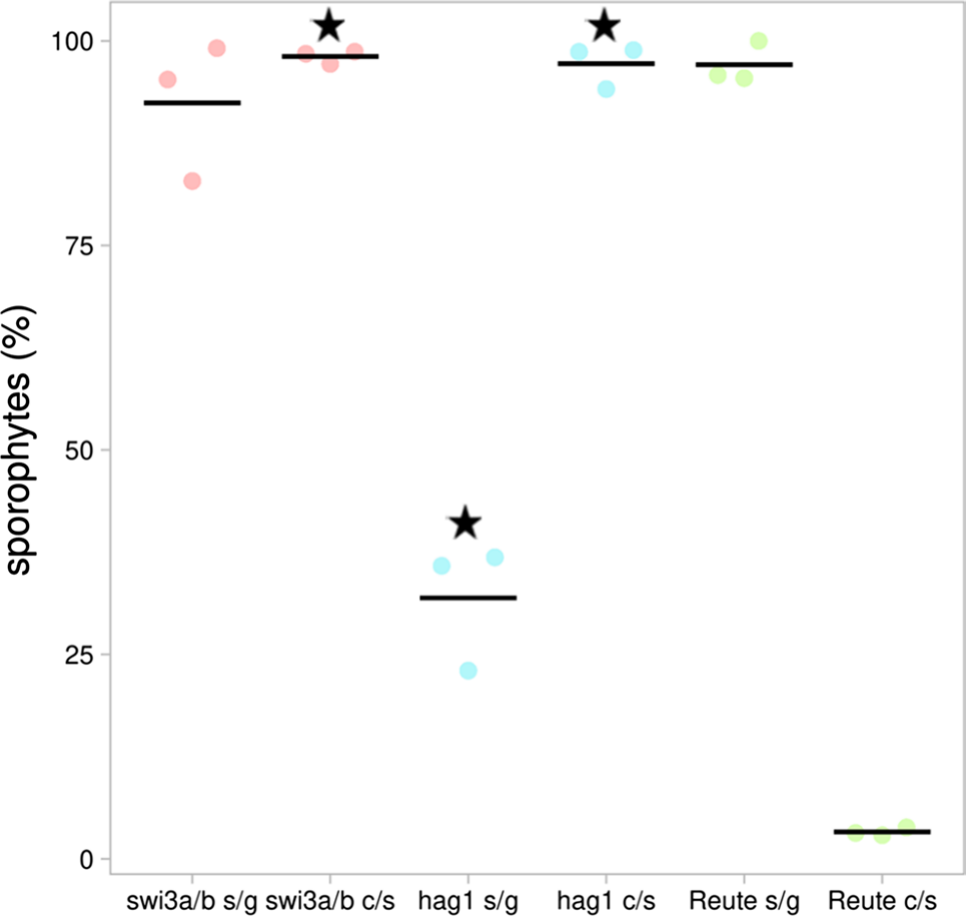
Crossing analyses with a fluorescent male fertile strain to test for male impairment. Pp*swi3a/b* and Pp*hag1* were crossed with Re-mcherry according to Perroud et al. (2019). Shown is the number of sporophytes per gametophore (s/g) in percentage relative to the total number of gametophores, and the rate of crosses per sporophytes (c/s) in percentage relative to the total number of sporophytes. Most of the sporophytes in the Reute control derive from selfing (homozygous; hence low number of heterozygous sporophytes, c/s). In contrast, almost 100% of mutant sporophytes are heterozygous (asterisks), indicating a male impairment (*p* < 0.01, Fisher’s exact test). The cross with the male fertile strain could largely restore the phenotype in *swi3a/b* (sporophyte/gametophore ratio at least 80%), while Pp*hag1* shows a significantly reduced sporophyte ratio (asterisk) as compared to the control. Three independent replicates (dots) were performed for the mutant lines as well as the control. The total number of gametophores analyzed was 621 (*swi3a/b*), 770 (*hag1*) and 190 (control). Averages of replicates are shown as horizontal lines. Numbers per individual mutant line are shown in Table S2

In order to determine the role of the male germ line, the mutants were crossed with a male fertile strain (Perroud et al. 2019) that served as sperm donor in order to fertilize the mutant egg cell. The control is highly self-fertile with an outcrossing ratio (heterozygous offspring) of 3%, and a sporophyte per gametophore development of 97% (Fig. 5), similar to previous studies (Meyberg et al. 2020; Perroud et al. 2019). Both mutants (*hag1* and *swi3a/b*) are male impaired, since almost 100% of developed sporophytes are crossed (heterozygous) offspring as compared to the highly self-fertile control (Fig. 5). *Swi3a/b* seems to be predominantly male impaired, since the phenotype can be almost fully restored by crossing with a male fertile partner (Fig. 5). The expression data indicate a gene function in sporophyte development as well (Fig. S4). This could not be proven in homozygous offspring because of the significant reduction of sporophyte development. With regard to the almost fully restored phenotype it can be hypothesized that the function in sporophyte development is recessive and can be complemented by a male fertile partner. This is not the case for *hag1*, which shows still a significant reduction of sporophyte development even when crossed with a fertile male partner (Fig. 5), indicating an additional impairment during fertilization or early embryogenesis. To elucidate whether what we consider crossed (heterozygous) offspring is not due to spurious selfing (fertilization by male sperm of the mutant), we crossed both *swi3a/b* and *hag1* with a male sterile mutant and found indeed that no sporophytes at all are formed (Fig. S8), demonstrating that the male germ line impairment is a dominant effect in both mutants.

**Fig. 6:**
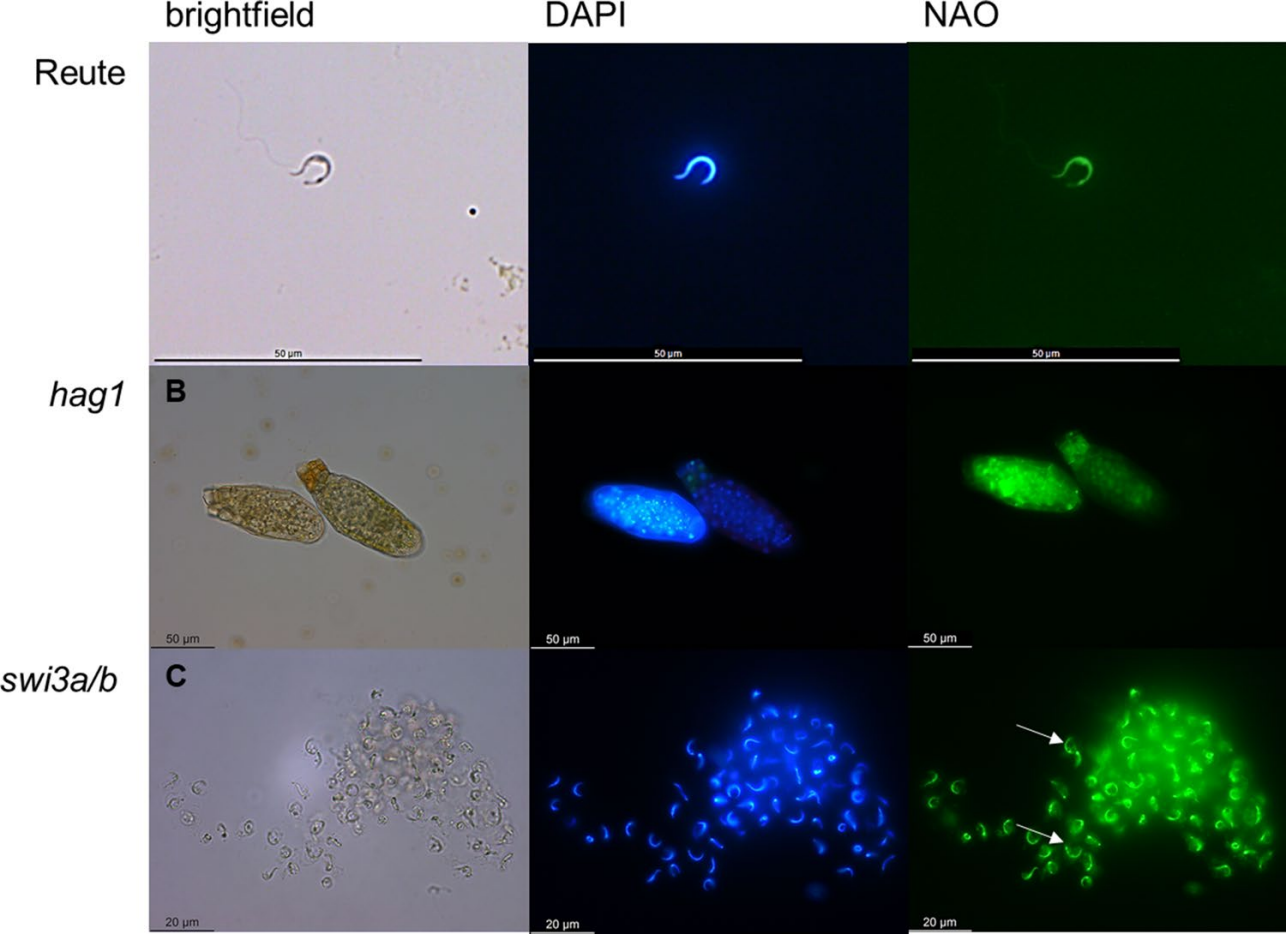
Spermatozoid analysis via a double staining with DAPI and NAO. A) A released Reute (control) spermatozoid is shown with its slender shape und fully reduced cytoplasm. B) Pphag1 antheridia do not release spermatozoids, but they remain in a round shape inside the antheridia. C) Ppswi3a/b antheridia release spermatozoids, which however show incomplete cytoplasmic reduction (white arrows). See Figs. S9-11 for further micrographs of sperm morphology

To further elucidate the nature of the male germ line impairment, spermatozoids were studied by double staining with DAPI and NAO (Fig. 6) to check for nuclear condensation as well as proper cytoplasmic reduction according to Sanchez-Vera et al. (2017). The spermatozoids of the control are characterized by a slender shape with fully reduced cytoplasm, closely surrounding the nucleus. Pp*swi3a/b* antheridia tip cells burst upon water uptake, comparable to the control. However, Pp*hag1* antheridia do not open and release their spermatozoids, but they remain as round-shaped structure inside the antheridia (Fig. 6). The arrest of antheridia development was further elucidated (see below, section Pp*hag1* impaired gametangia maturation); the analyses of different time points show clearly that the defect is due to an arrest but not a delay. The NAO staining revealed for Pp*swi3a/b* an impairment of cytoplasmic reduction (Figs. 6, S11). Since Pp*hag1* does not properly release spermatozoids from its antheridia, and Pp*swi3a/b* seems to be impaired in the latest steps of spermatozoid ripening, we focused on gametangia development and spermatozoid movement/structure, in subsequent analyses.

### Pp*swi3a/b* spermatozoids are impaired during late maturation

**Fig. 7:**
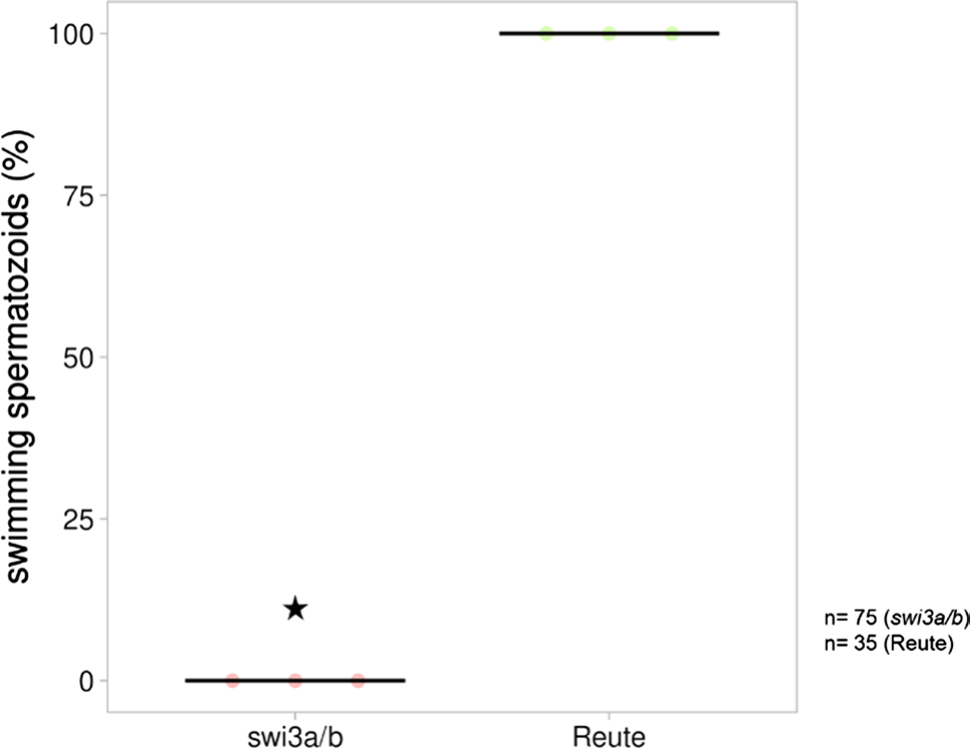
Swimming capability of spermatozoids shortly after release. Mature antheridia (21 dpi) release their spermatozoids, which were analyzed in terms of movement in three independent replicates in Ppswi3a/b and the control. The released spermatozoids of each antheridium were analyzed in terms of movement. The movement was classified as swimming and no swimming. Swimming was defined as the ability to move away independently from the position of antheridial release. The control showed motile, swimming spermatozoids in 100% of analyzed antheridia, whereas no swimming at all was observed in Ppswi3a/b (significant reduction, *p* < 0.01, Fisher’s exact). In total, 75 (*n* = 29) (first replicate), 32 (second replicate), 14 (third replicate) antheridia (Ppswi3a/b) were analyzed that opened spontaneously. For the control, 35 antheridia (*n* = 10, 17 and 8 for the first, second and third replicate, respectively) were analyzed. Averages of replicates are shown as horizontal lines

Spermatozoid analysis of Pp*swi3a/b* 21 d after induction of gametangiogenesis showed that the spermatozoids are impaired in their movement (Fig. 7). The swimming capability was analyzed in mature antheridia bundles (21 days post induction, dpi). The spermatozoids of the control started swimming shortly after release, and 100% were able to swim. With regard to Pp*swi3a/b*, no spermatozoid was observed that was able to swim. In total, 28% of sperm masses were gently shaking after release, while the remaining 72% of sperm masses did not move after release.

**Fig. 8:**
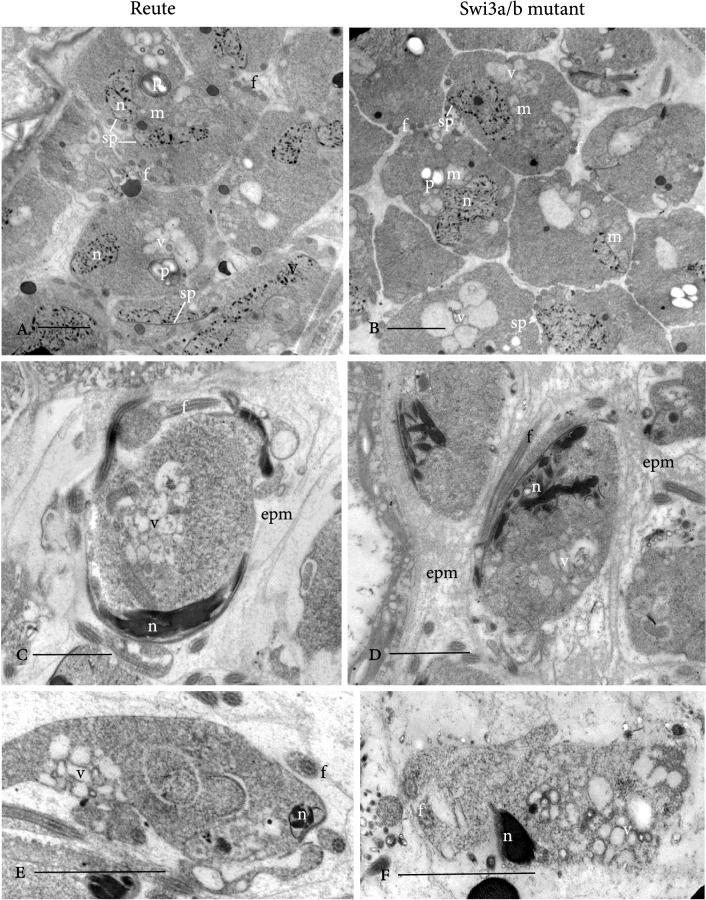
TEM of developing spermatids. Reute control in left column and Pp*swi3a/b* mutant in right column. **a**, **b** Identical early stages of spermatid differentiation reveal elongating flagella (f), a microtubular spline (sp) and associated condensing nucleus (n), a single starch-laden plastid (p) with mitochondrion (m). Vacuoles and vesicles (v) are evidence of cytoplasmic reduction. **c**, **d** As nuclear compaction nears completion, deviations from Reute control (C) to Ppswi3a/b gametes (D) include irregular chromatin condensation and an abnormal extraprotoplasmic matrix (epm) in which gametes develop that consist of dense material and anastomosing fibers different from the fine granular to electron-lucent matrix around Reute gametes. **e**, **f** A central network of coated and smooth vesicles (v) is evidence of continued cytoplasmic reduction in both Reute (**e**) and Pp*swi3a/b* (**f**) gametes. Bars: A-F = 2 mm

**Fig. 9:**
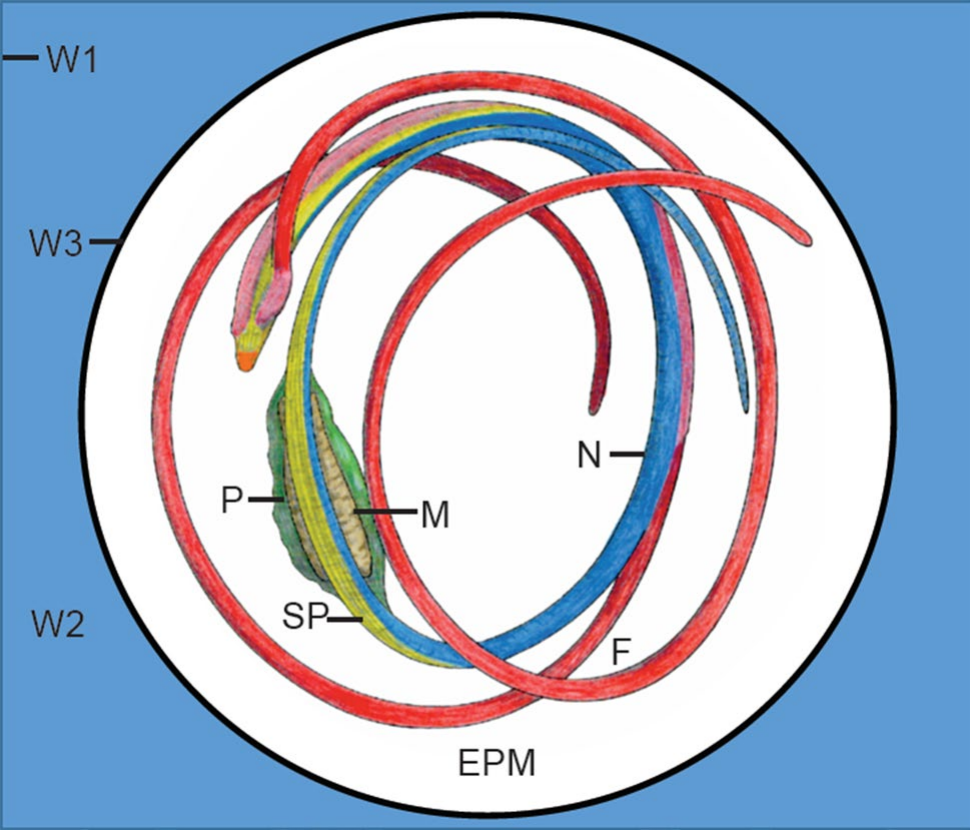
Cartoonized schema of a developing sperm cell with focus on cell walls. Spermatogenesis in moss involves the deposition of four sequential cell walls or matrices. Wall one (W1) is the primary cell wall stemming from antheridial cell divisions. The thick blue wall (W2) is deposited and makes the cell round/spherical. The black inner line around the spermatozoid is the third wall (W3) or "vesicle" (cf. Figs. 8/10) in which gametes are released. The fourth wall layer is the extraprotoplasmic matrix (epm) in which the sperm cells develop. Each wall has a different polymer composition, and only W3 remains intact when gametes are mature/released. Mature wildtype gametes have two anterior flagella (red, f) that encircle the cell, an elongated cylindrical nucleus (blue, n), a spline (sp) of microtubules that forms the backbone of the cell and two mitochondria (brown, m), one at the anterior (not visible) and one in the mid-region of the nucleus that is associated with a single plastid (green, p). In Pp*swi3a/b* gametes, extraneous cytoplasm does not break down and the wall layers are not properly degraded, leaving the gametes embedded in a cloud of fluorescent material. The caviar-like structure of Pp*swi3a/b* (cf. Figs., S9, S11) is likely the outline of wall 3 due to incomplete degradation of cell wall polymers and the retention of remnants of each of the four wall layers

**Fig. 10:**
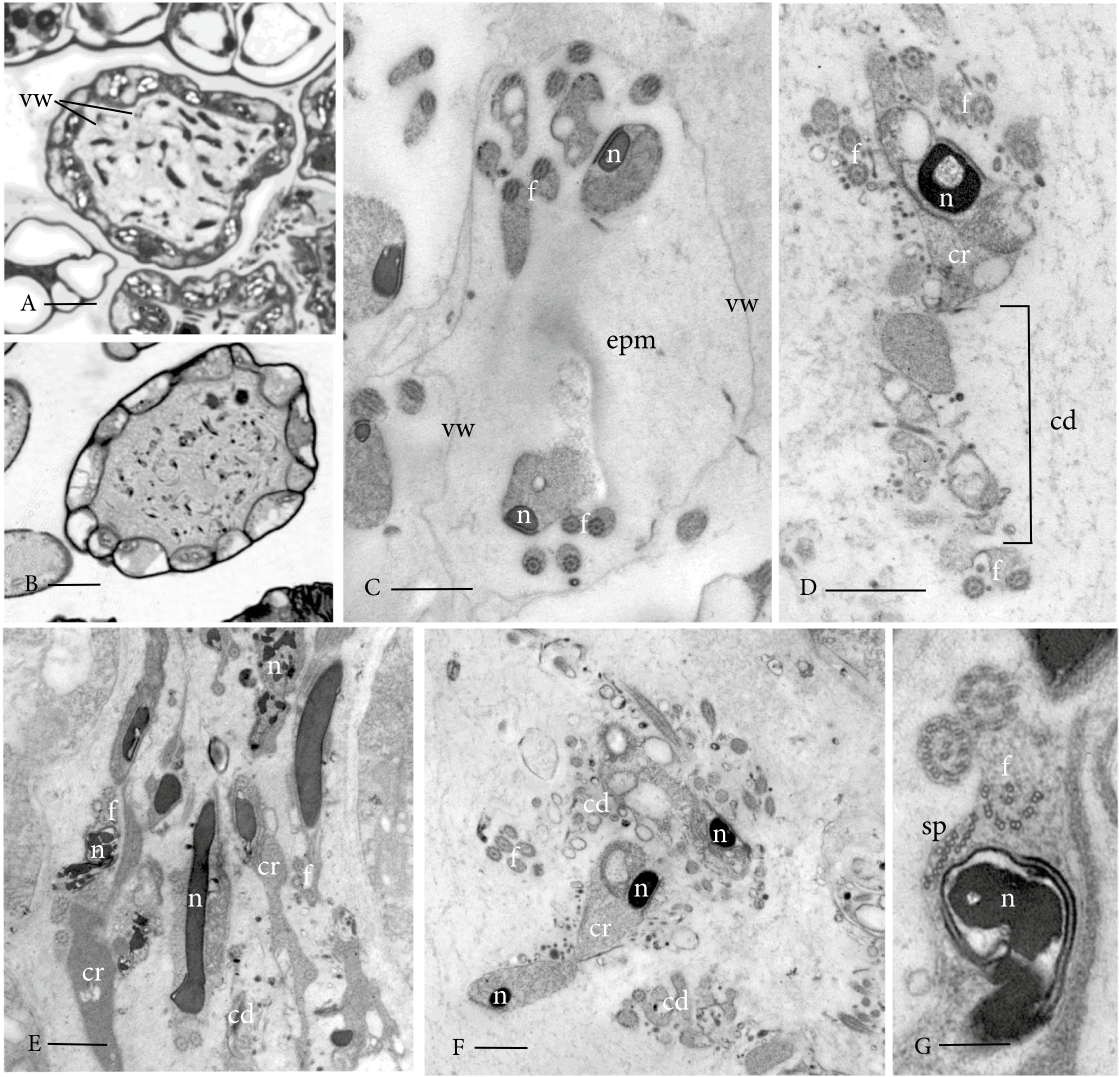
Mature antheridia and gametes. **a** LM cross section, Reute (control) antheridium contains evenly spaced, streamlined, coiled gametes individually enclosed in a round/spherical thin “vesicle” wall (vw). **b** Ppswi3a/b gametes are poorly defined and embedded in a dense irregular matrix. **c** TEM of nearly mature Reute sperm cell showing two profiles of the coiled nucleus (n) and flagella (f) embedded in the extraprotoplasmic matrix (epm) that is electron-lucent and enclosed in a “vesicle” wall (vw) or third wall; cf. figure **d**–**g** TEMs of Pp*swi3a/b* gametes showing disrupted cell shape, irregular nuclear (n) compaction and coiling, abnormal locomotory apparatus and cytoplasmic remnants (cr) attached to the cell body. The matrix in which gametes are embedded is irregularly granular/fibrillar with abundant cytoplasmic debris (cd). The “vesicle” wall is typically not delineated but may remain in less severe phenotypes. **g** Disrupted spline (sp) and malformed axonemes (f) are common in the mutant. Bars: A, B = 10 mm; C, D, E, F = 1 mm; G = 0.25 mm

The movement impairment might be due to an enclosure in caviar-like structures and incomplete cytoplasmic reduction (Figs. 6, S9, S10, S11, S12, S14). These two phenomena reflect two observable aberrations as different manifestations of the same defect. In the control, the sperm cell cytoplasm is surrounded by an extracellular matrix in which the gametes develop; this matrix is broken down during differentiation, enabling sperm cells to swim when released (Lopez and Renzaglia 2014). The mutant exhibits a persistent extraprotoplasmatic matrix inside the “vesicle” wall (cf. Figs. 8, 9, 10), indicating impairment of sperm cell development. Recently, it was shown that autophagy is essential for *P. patens* gamete differentiation in terms of cytoplasmic reduction (Sanchez-Vera et al. 2017). The caviar-like structure (Fig. S11) could be seen in 37.8% of the analyzed pictures, whereas incomplete cytoplasmic reduction/breakdown of cell wall material was observed in 48.8% of analyzed pictures (Fig. S11). An overlap of caviar-like structures and incomplete reduction was observed in 13.5% of analyzed pictures.

### Pp*swi3a/b* spermatozoids show ultrastructural differences during maturation

In order to determine ultrastructural details of sperm maturation, samples were embedded, stained and analyzed by light and transmission electron microscopy. No differences were apparent between mutant and control plants in antheridial development up to the establishment of spermatids (undifferentiated sperm cells; Fig. S13).

The origin and early development of the locomotory apparatus are similar in Reute and *Pp*swi3a/b spermatids. The locomotory apparatus begins to form in the young spermatid and consists of two basal bodies, from which the flagella develop, and an underlying lamellar strip and anterior mitochondrion. A microtubular backbone, the so-called spline, grows around the outside of the cell, and the nucleus elongates and condenses down along it (Fig. 8a, b). Chromatin condensation begins with irregular strands in a random arrangement. A single starch-laden plastid and associated mitochondrion attach to the central part of the nucleus, and an internal membrane system of vacuoles and vesicles provides the first evidence of the elimination of extraneous cytoplasm (Fig. 8a–d). As nuclear compaction nears completion, deviations of *Pp*swi3a/b spermatid development from the wildtype cells are evident (Fig. 8c, d): condensation of chromatin is orderly in wildtype spermatids (Fig. 8c) and is often irregular in *swi3a/b* (Fig. 8d). Evidence of cytoplasmic reduction is seen in both, mutant and control, in the form of a central network of coated and smooth vesicles (Fig. 8e, f). The most striking difference is in the matrix surrounding developing spermatids. It is in this matrix that flagella elongate, the cell changes shape and the excess cytoplasm is deleted. Around Reute gametes, the wall layers are distinct and the cell wall matrix is fine granular to electron-lucent (Fig. 8c). In Pp*swi3a/b*, the matrix consists of a network of anastomosing fibers that persist throughout development (Fig. 8d). The demarcation between wall layers in the mutant is blurred. In Reute spermatids, as generally in differentiating motile gametes of mosses, there are four sequential wall layers that formed during differentiation (Fig. 9). Each layer has a unique composition of cell wall polymers that include cellulose, pectins, hemicelluloses and arabinogalactan proteins (Lopez et al. 2017; Lopez-Swalls 2016).

Within nearly mature unopened Reute antheridia, the coiled gametes are individually enclosed in spherical thin “vesicle” walls (3rd wall in Fig. 9) that are evenly spaced within a dense matrix (Fig. 10). Cell wall materials degrade prior to dehiscence except the “vesicle” in which spermatozoids are released. In contrast, the most severely disrupted *Pp*swi3a/b gametes are unevenly separated within an ill-defined, fragmented reticulum (Fig. 10B-D). In cross section, the nearly mature Reute gamete is streamlined with little remnant cytoplasm and enclosed in the “vesicle” wall (3rd wall) (Figs. 9, 10c, S14A, C). In Pp*swi3a/b* gametes, the cytoplasm is incompletely eliminated and multiple cytoplasmic remnants remain attached to cells that are surrounded by a matrix containing cytoplasmic debris (Figs. 10d–f, S14B, D). Cells may or may not be coiled and nuclei are often incompletely and unevenly compacted (Figs. 10d, e, g, S14B, D). The locomotory apparatus may be disrupted, and axonemes are often malformed (Figs. 10g, S14D). In less severely disrupted Pp*swi3a/b* sperm cells the wall layers are not completely degraded and the rounded thin “vesicle” wall remains around the disrupted extraprotoplasmic matrix (Fig. 9). This likely explains the caviar-like appearance that is observed by fluorescence microscopy (Fig. S11).

In summary, Pp*swi3a/b* spermatozoids fail to correctly degrade the series of wall layers that are produced during spermatogenesis. Likewise, the cytoplasm around the nucleus and outside of the cell is not broken down in a normal fashion, even though the mid-stage gametes produce an internal membrane system of vesicles for cytoplasmic elimination similar to that in Reute and mosses in general (Miller and Ducket 1986; Bernhard and Renzaglia 1995) around the nucleus. Unlike Reute spermatozoids, that are free from any extracellular matrix at maturity, the mature Pp*swi3a/b* sperm cells are surrounded by fragmented cytoplasm and remain embedded in the partially broken-down cell wall matrix.

### Pp*hag1* impaired gametangia maturation

Testing for male impairment showed not only that *hag1* is not able to fertilize, but crossing with a male fertile strain cannot fully restore the phenotype; the number of sporophytes is still significantly reduced (Fig. 5). This shows that *hag1* is not only male impaired, but the reduced sporophyte development indicates that either the female gametangia development is impaired or the defect affects the fertilization and/or post fertilization.

The gametangia are formed on the tip of the gametophore. The male sexual organ is called antheridium and develops from an antheridium initial stem cell (Kofuji et al. 2018a). A mature apex develops a bundle of antheridia. Antheridia comprise a single outer cell layer that encapsulates the motile spermatozoids. Through a droplet of water, the swollen antheridia tip cell bursts and releases the flagellated gametes, which are able to swim. Shortly after initiation of antheridia development, archegonia development starts. Archegonia are the female gametangia, which are flask shaped. The egg develops inside the archegonial venter. To achieve fertilization, the spermatozoids swim toward the archegonium and enter the opened archegonial neck and then swim through it to reach the egg cell (Hiss et al. 2017; Landberg et al. 2013; Sanchez-Vera et al. 2017; Kofuji et al. 2018b).

**Fig. 11:**
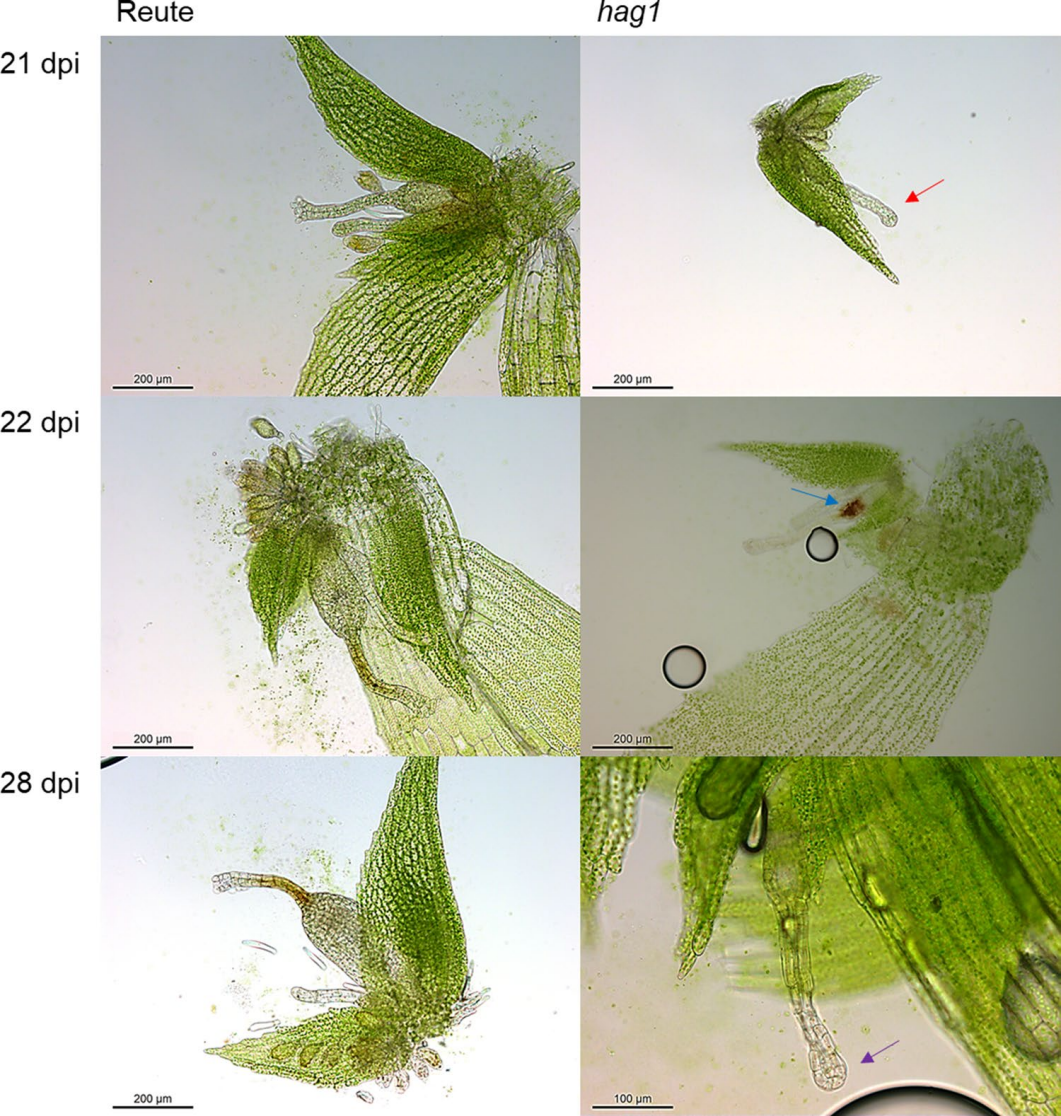
Archegonial development at 21, 22 and 28 dpi. Reute (control) archegonia are open at 21 dpi (**a**) and fertilized at day 22 (**b**) (embryo development), at day 28 (**c**) a pre-meiotic early sporophyte has developed (Hiss et al. 2017). In contrast, hag1 archegonia in many cases do not open (red arrow) but develop a brownish coloration (blue arrow) or bleaching (purple arrow) 22 and 28 dpi. As soon as an analyzed antheridium or archegonium was classified as opened, the apex was not further analyzed (characterized as mature). In terms of closed gametangia, no open antheridium or archegonium was found onto the respective apex. The timepoints were chosen according to Hiss et al. (2017)

**Fig. 12:**
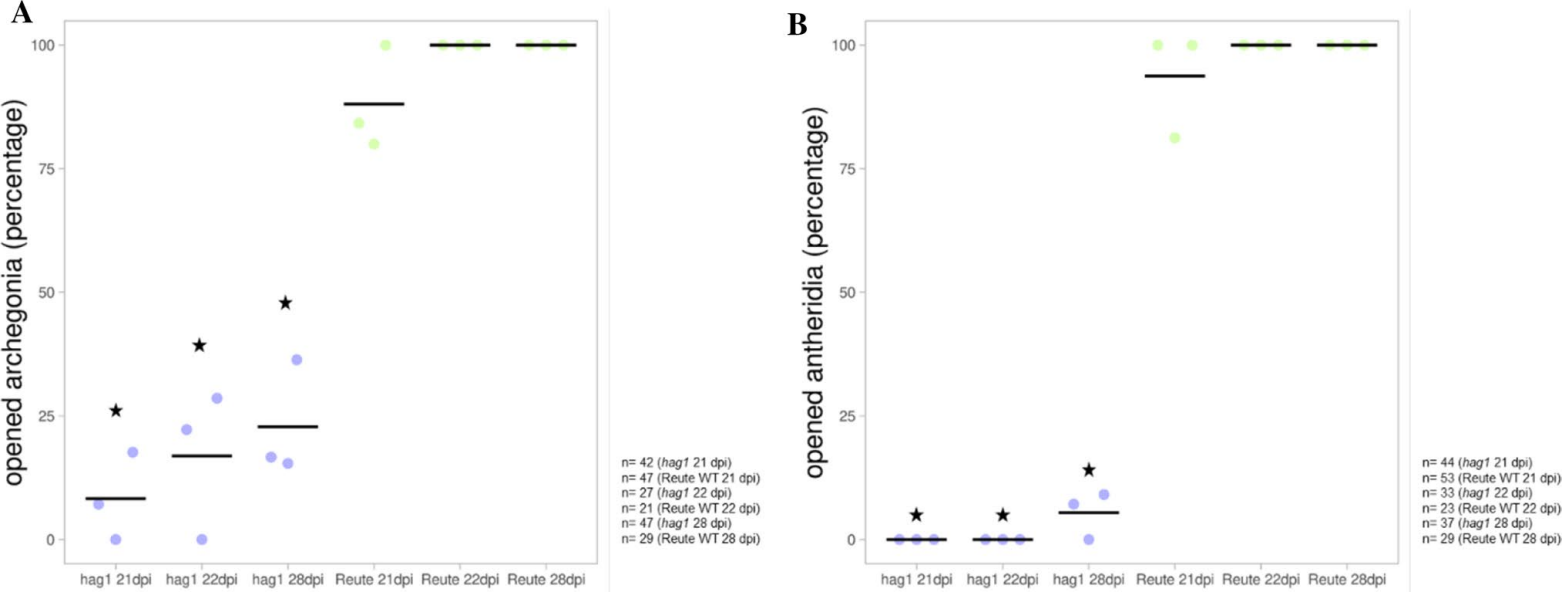
Analyses of opened Pp*hag1* archegonial neck cells and antheridia. A The archegonia of the Reute control as well as Pp*hag1* were analyzed in terms of archegonial opening 21, 22 and 28 dpi in three independent replicates. Pp*hag1* shows a significantly lower (asterisks) opened archegonia rate at every analyzed time point compared to the respective control (*p* < 0.05, Fisher’s exact test). The total number of analyzed archegonia per mutant line/control at each time point is shown to the right. The number of opened archegonia was calculated as percentage relative to the total number of counted archegonia. Averages of replicates are shown as horizontal lines. B The antheridia of the control as well as Pphag1 were analyzed 21, 22 and 28 dpi in terms of antheridial opening in three independent replicates. Pphag1 shows a significantly lower (asterisks) number of opened antheridia at every analyzed time point compared to the control (*p* < 0.01, Fisher’s exact test). The total number of analyzed antheridia per mutant line/control at each time point is shown to the right. The number of opened antheridia was calculated in percentage relative to the total number of counted antheridia. Averages of replicates are shown as horizontal lines

To further clarify the nature of the impairment, archegonial development was analyzed 21, 22 and 28 dpi (Fig. 11). Archegonia of the control open 21 dpi and develop sporophytes after self-fertilization 22 and 28 dpi. Pp*hag1* shows archegonia which are still closed at the described timepoints. The archegonia at 22 dpi exhibit a brownish coloration, and the archegonia at 28 dpi are discolored. The number of opened archegonia was counted in the control as well as the mutant at the three described timepoints (Fig. 12a). In the control 88% of archegonia were open at 21 dpi and 100% of archegonia open or already fertilized at 22 and 28 dpi. Pp*hag1* shows a significantly reduced number of 8% of opened archegonia 21 dpi, 1% at 22 dpi and 23% at 28 dpi.

As outlined above, Pp*hag1* antheridia do not open spontaneously. Antheridia of the control opened to 94% at 21 dpi and 100% at 22 and 28 dpi. Pp*hag1* antheridia, however, did not open at 21 and 22 dpi, and only 5% of antheridia were open at 28 dpi (Fig. 12b). Also, antheridia bundles showed a brownish coloration as well as bleaching, those antheridia that opened released round, shapeless spermatozoid agglomerates (Fig. S15).

The mutant line analyzed here showed a sporophyte/gametophore ratio of 13% (Fig. 5), which is in the range of the number of ripe/opened archegonia 21 dpi (8.3%, Fig. 12a). Hence, the observed reduced sporophyte rate even when crossing with a male fertile partner may well be due to the disturbed archegonial maturation. Archegonia phenotyping results 28 dpi (Fig. 11) show that female gametangia ripening is not only delayed but arrested. The archegonia turn brown and colorless, indicating premature senescence. Premature senescence was shown to be autophagy-related (Sanchez-Vera et al. 2017). Also, we observed archegonia which were open 21 dpi, but showed a brownish coloration around the egg cell (Fig. 11), indicating a disturbance in development as well. As described above, a brownish coloration of the canal cells develops upon entrance of a spermatozoid (Tanahashi et al. 2005). The coloration observed here can be observed in the canal cells despite lack of fertilization (Fig. 4), as well as surrounding the egg cell (Fig. 11).

### Mp*hag1* fails to initiate gametangiophores in both sexes

**Fig. 13:**
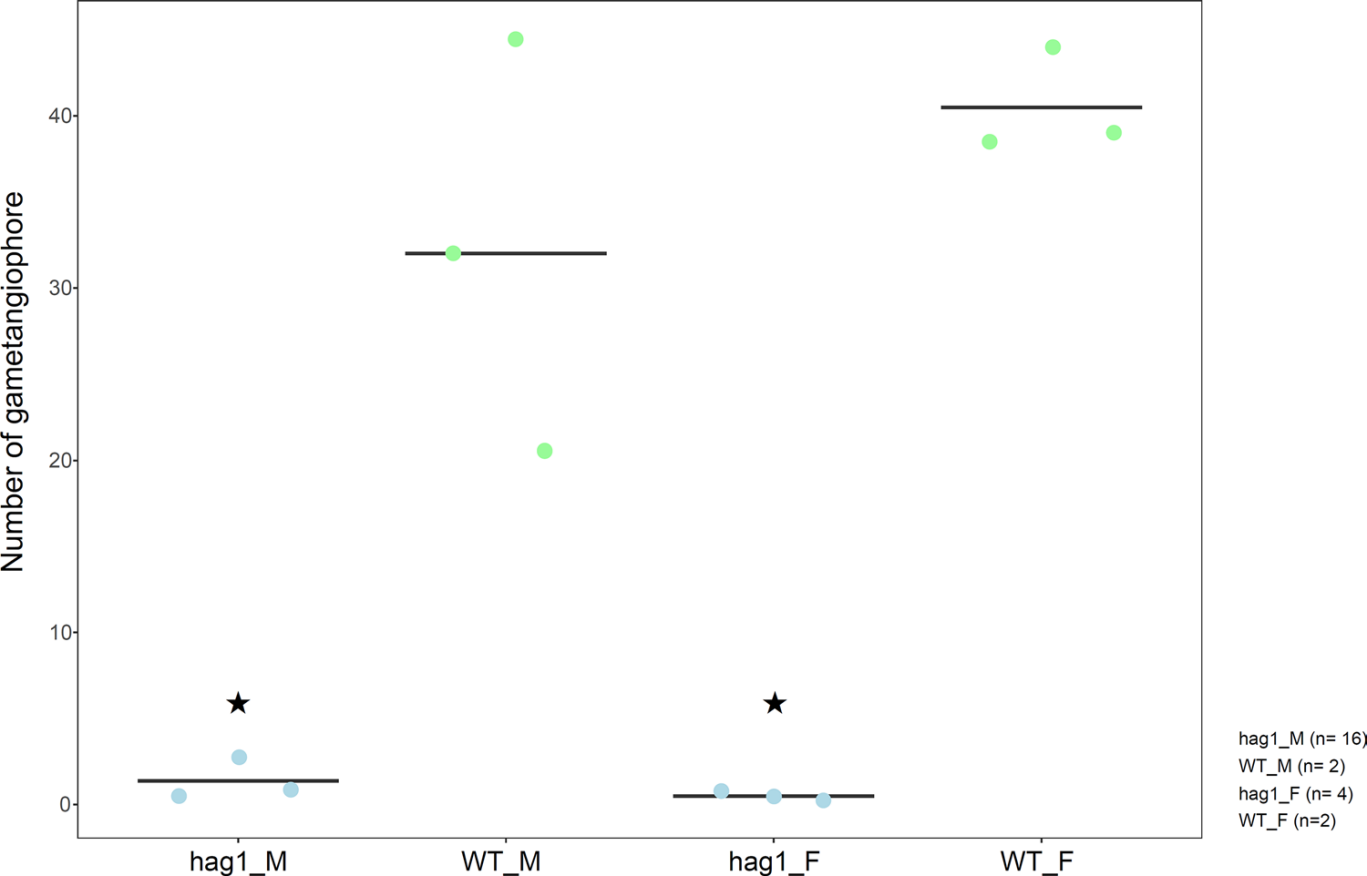
Gametangiophore development of Mp*hag1*. Three independent replicates (dots) were analyzed. Green dots represent wild-type (control) lines, whereas blue dots represent mutant lines (m = male, f = female). Averages of replicates are shown as horizontal lines. The mutants develop significantly less gametangiophores per plant as compared with the control (*p* < 0.05, *t* test). See Table S6 for details per mutant line

In Pp*swi3a/*b there is a clear defect late in male germ line maturation. However, Pp*hag1* shows comparatively early defects in the male and female gametangia development. *P. patens* is a monoecious plant, in which male and female gametangia develop from the same plant. We were interested to see whether the early effect on gametangia is separated by sex and hence analyzed the gene function in a dioecious bryophyte, the liverwort model *Marchantia polymorpha*. Gametangiophores are Marchantiales-specific structures that bear male and female gametangia. In *P. patens*, the gametangia develop directly from gametophores. The commonality between gametangiophores and gametangia is that both are structures that develop for sexual reproduction. In both cases, they develop from vegetative tissue (Hiss et al. 2017; Shimamura 2016). The male and female CRISPR-Cas9 loss-of-function mutants were genotyped (Fig. S16) and analyzed in terms of gametangiophore formation. Strikingly, both male and female Mp*hag1* developed significantly less gametangiophores than the control (Fig. 13).

Apparently, the expression of the genes that control the development of male and female reproductive organs is activated during gametogenesis in *P. patens*, whereas expression differences are already in place in the male/female vegetative thallus of *M. polymorpha*. Loss-of-function mutants in both species show a significant reduction of sexual reproduction. Contrary to *P. patens*, the Mp*hag1* leads to an earlier defect, namely disturbed emergence of gametangiophores (that carry the gametangia).

### RNA-seq of the *P. patens* mutants confirms lack of fertilization

RNA-seq analyses were carried out to identify the involvement of the candidate genes in the transcriptional network during gametangia ripening as well as the initiation of embryogenesis. Gametophore apices (containing the gametangia) were harvested for RNA isolation 21 dpi as well as two days after watering (2 daw = 23 dpi). 21 dpi, the control develops ripe gametangia. Fertilization can be synchronized via watering and has occurred 2 daw so that the gene regulatory program of embryogenesis should have been initiated. In both mutants (Pp*swi3a/b* and Pp*hag1*) self-fertilization is not possible, leading to an arrest in the life cycle during gametangia development. Hence, we expected to see transcriptional differences between control and mutants.

In Pp*swi3a/b* 771 uniquely upregulated DEGs as compared to the control were detected upon fertilization (2 daw), and only 14 at 21 dpi, in the absence of fertilization (Fig. S18). The low number of DEGs prior to fertilization indicates that there are no major differences in the transcriptional network during gametangia ripening. These results reflect the mutant phenotype in the sense that Pp*swi3a/b* develops wild-type-like archegonia, whereas the male germ line is impaired in the latest steps of spermatozoid ripening, so that no major differences in the transcriptional network in gametangia ripening are observable. In contrast, 2 daw the high number of DEGs might reflect the maintained gametangial program in Pp*swi3a/b* (since no fertilization occurs), in contrast to the control that 2 daw already started the zygotic transcriptional program.

To further elucidate the transcriptional response, DEGs were characterized via their annotation and expression profiles plotted (Fig. S19). In terms of late embryogenesis abundant (LEA)/embryogenesis-associated genes we found that some transcripts are less abundant in both mutants than in the control, while others are more highly abundant in both mutants than in the control. Of interest is Pp3c3_14110V3.1, a receptor kinase that is downregulated in both mutants and hence might be involved in activating embryogenesis. Before watering, flagella-associated transcripts are generally less abundant in the HAG1 mutant than in both the control and the SWI3 mutant. Potentially these genes are not activated by K14 acetylation during gametangiogenesis. Several transcripts are more abundant in the SWI3 mutant than in the control, both before and after watering, indicating abolished regulation by the SWI3 complex. The same pattern is somewhat reflected by the chromatin-associated DEGs. Some of them are less abundant in the HAG1 mutant than in the control, and more highly abundant in the SWI3 mutant. This could reflect a lack of activating histone acetylation (HAG1), respectively, a lack of repressing the PRC2 complex by SWI3 and hence lack of deactivating K27me3 marks.

Interestingly, the AtEMS1 ortholog Pp3c17_21540V3.1 shows lower abundance in Pp*swi3a/b* (before as well as after watering) than in the control. *Ems1* mutants in *A. thaliana* are male sterile due to a failure in microsporogenesis (the mutant suffers from excess microsporocytes and a lack of tapetal cells and does not break down the middle layer cells). The meiotic nuclear division takes place, but cytokinesis is not performed, resulting in abnormal microsporogenesis leading to male sterility. The gene *EMS1* is encoding a putative leucine-rich-repeat receptor kinase (LRR-RK) (Zhao et al. 2002). The lower expression of *EMS1* in Pp*swi3a/b* and At*ems1* causes a comparable phenotype, indicating a similar role of ems1 orthologs in bryophytes and angiosperms. It can be hypothesized that the lower expression of *EMS1* in Pp*swi3a/b* is due to a lacking regulatory effect due to the *SWI3A/B* loss of function. In addition, PACRG is upregulated in the mutant; in mice the ortholog is highly expressed in testes during spermatogenesis and the gene product is abundant in mature sperm. Mutations lead to malformations in the sperm head as well as defects in the flagellum (Lorenzetti et al. 2004). It was shown that PACRG is involved in flagellar tubulin binding (Dawe et al. 2005).

In Pp*hag1* 294 uniquely upregulated DEGs were detected 2 daw, compared to 30 at 21 dpi without watering, which again indicates that the gametangial transcriptional program at 2 daw still is activated, since no fertilization occurs. We find lower abundance of Pp3c23_9320V3.1, an ortholog of AtTRAUCO, in *hag1*. In *A. thaliana* the transcript levels show a peak in pollen, sepals, anthers and seeds. The presence of transcript levels in the respective tissues indicates a role in flowering development. Mutations of At*TRAUCO* in heterozygous plants show an embryo-lethal phenotype, since approximately 25% of seeds abort. It can be hypothesized that there is a paternal defect which cannot be proven, since homozygous embryos abort. This is comparable to Pp*hag1* since sporophytes do not develop throughout selfing. Therefore, the gene function could not be analyzed in sporophytes. The lower abundance in *hag1* mutants in *P. patens* indicates a similar role as in *A. thaliana trauco* mutants (Aquea et al. 2010). In addition, it can be speculated that the lower abundance of Pp3c23_9320V3.1 in Pp*hag1* compared to the wild type is caused by the missing regulation due to the loss of function. The hypothesized paternal role of TRAUCO in *A. thaliana* can be substantiated by the *P. patens* phenotype, namely a delay in gametangia ripening and an arrest at premature stages, indicating a conserved role of the described gene network.

### Evolutionary conservation of (male) germ line control

#### SWI3

Chromatin-remodeling complexes (CRCs) constitute a switch point between signaling pathways and chromatin-based control of transcription. They are involved in transcriptional activation as well as repression (Sarnowski et al. 2005; Roberts and Orkin 2004). AtSWI3A/B (AT2G47620) is part of the SWITCH/SUCROSE NONFERMENTING (SWI/SNF) CRC, which is involved in ATP-dependent alteration of DNA-histone contacts (Sarnowski et al. 2005). The genome of *A. thaliana* encodes four SWI3-like proteins, which are diverse in their function. Mutants At*SWI3A* and At*SWI3B* have severe defects in embryo development, causing a lethal arrest at the early globular stage (Sarnowski et al. 2005).

In *A. thaliana* mutations in *SWI3A* or *B* lead to a block of embryo development at the globular stage. The embryo lethal phenotype results in white, aborting seeds in homozygous plants after self-pollination. Insertion mutant alleles lead to a recessive embryo lethal phenotype, while homozygous mutants were not viable. These seeds show delayed development, and the embryos are arrested in the late globular stage. These findings indicate that AtSWI3A/B plays an important role in early embryo development (Sarnowski et al. 2005). The mutant phenotypes in *A. thaliana* (angiosperm) and *P. patens* (bryophyte), arrest of sexual reproduction, indicate an evolutionary conserved regulatory function of the gene in sexual reproduction/embryogenesis.

A. *thaliana* encodes four SWI3 proteins, which are diverse in their function. Beyond *swi*3a and b leading to an arrest of embryo development, *swi*3b mutants exhibit an impairment in macrosporogenesis (arrested ovules) as well as microsporogenesis (Sarnowski et al. 2005). With regard to the failure of macrosporogenesis, it was shown that megaspores can develop normally in the ovules after meiosis, but roughly 50% of ovules did not form embryo sacs, so that the megaspores failed to divide subsequently, but instead stayed in an arrested central position. A mutation in At*SWI3b* caused partial male sterility. A deeper analysis of microsporogenesis revealed that pollen mother cells underwent meiosis, followed by callose-separated tetrads and divided vacuolated microspores. Nevertheless, about 50% of the microspores did not undergo further divisions but arrested, vacuolated and were exposed to senescence, which leads to lipid deposition and organellar degradation or collapsing (Sarnowski et al. 2005). Pp*swi3a/b* shows a strong male impairment reminiscent of the *A. thaliana swi*3b phenotype.

A previous phylogenetic analysis suggested that of the four *SWI3* genes in *P. patens* one is an ortholog to At*SWI3a*, whereas the remaining three are paralogs of At*SWI*3c (Gao et al. 2012). Our data, however, show that the *P. patens* gene formerly designated as Pp*SWI*3*a* is sister to the At*SWI3a/b* clade (Fig. 2). This finding is likewise true for the other bryophyte lineages. The three remaining Pp*SWI3* paralogs are part of the *SWI3c/d* clade, which probably represents the ancestral gene. A duplication event apparently gave rise to the land plant-specific a/b clade that further diversified during seed plant evolution. The ancestral function for SWI3A/B might have been the control of male germ line maturation. The function diversified in seed plants after acquisition of the a and b paralogs, covering male and female sporogenesis as well as embryogenesis.

#### HAG1

Analyses of Pp*hag1* antheridia revealed that maturation is disturbed following formative cell divisions, and the establishment of rounded nascent spermatids occurs much earlier than in Pp*swi3a/b*. At 21 dpi antheridia are not open and developing spermatozoids are round, bulky and do not condense toward a slender shape. Antheridia turn completely brownish without opening 28 dpi, which might indicate that they are disturbed in apoptosis prior to maturity (Fig. S15). This would fit with the phenotype of the *GCN5* (the ortholog of *HAG1* described in mice) mutant, which shows embryonic lethality and an increased apoptosis (Phan et al. 2005). In *Mus musculus*, it was shown that a disruption of *GCN5* leads to a lethal embryonic defect (Xu et al. 2000; Yamauchi et al. 2000), indicating functional conservation in different eukaryotic lineages. These *gcn5* null embryos are not viable, since they exhibit increased apoptosis, which is restricted primarily to the mesodermal and ectodermal lineages (Phan et al. 2005; Xu et al. 2000). On top of that, it was shown recently that GCN5 is also essential for proper spermiogenesis in mice. The loss of *GCN5* leads to altered chromatin structures and an increase in histone retention in sperm, which then results in malformation (abnormal nuclear development) and disrupted male fertility in mice (Luense et al. 2019). Interestingly, the mouse phenotype resembles the moss phenotype with regard to sperm morphology.

The *A. thaliana HAG1* ortholog AT3G54610 (Kim et al. 2015), also known as GCN5, was shown to be predominantly involved in the acetylation of H3K14 (Benhamed et al. 2006; Earley et al. 2007; Mahrez et al. 2016). As a fundamental regulator, HAG1 is associated with a high number of developmental processes (Aquea et al. 2017), e.g., timing of juvenile-to-adult phase transition (Kim et al. 2015), inflorescence meristem and stamen development (Cohen et al. 2009), and miRNA production (Kim et al. 2009). Mutations in AtHAG1 are associated with the alternation of generations, namely abnormal flower development and reduced fertility (Bertrand et al. 2003; Vlachonasios et al. 2003). The loss of fecundity was attributed to a reduced stamen height in early-arising flowers and an increase in stamen number in early-arising flowers in contrast to later flowers that are able to produce small siliques with a few seeds (Vlachonasios et al. 2003). This loss of fecundity due to male reproductive tissue in flowering plants is comparable to the defect in the male germ line in *P. patens* mutants. Reproductive tissues in the flower can be considered equivalent to the gametangia in bryophytes (Rensing 2016). Recently, it was shown that At*clavata1*/*hag1* double mutants exhibit elongated gynoecia with reduced valves and enlarged stigma and style, leading to the hypothesis that CLAVATA signaling and GCN5 regulation play a synergistic role throughout gynoecium development. Single mutants of *gcn5* showed a milder phenotype in terms of gynoecium development (Poulios and Vlachonasios 2018). As shown here, mutations in *P. patens* also result in reduced fertility, primarily due to defects in the male germ line. However, mutants show an impairment in the female germ line as well, which is comparable to the *A. thaliana* mutant phenotype. *P. patens hag1* reflects the milder phenotype in the female reproductive development in single mutants of *gcn5* in *A. thaliana* (as compared to the At*clavata1*/*hag1* double mutant)*.*

Given the phylogenetic analysis (Fig. 1), *HAG1* shows a strong propensity not to be retained after paralog acquisition. This is probably due to dosage imbalance and suggests crucial interaction with partners, potentially such as CLAVATA. The function of HAG1 to control the formation and maturation of germ line-bearing tissues has probably been conserved since before the split of plants and animals.

## Conclusions

**Fig. 14:**
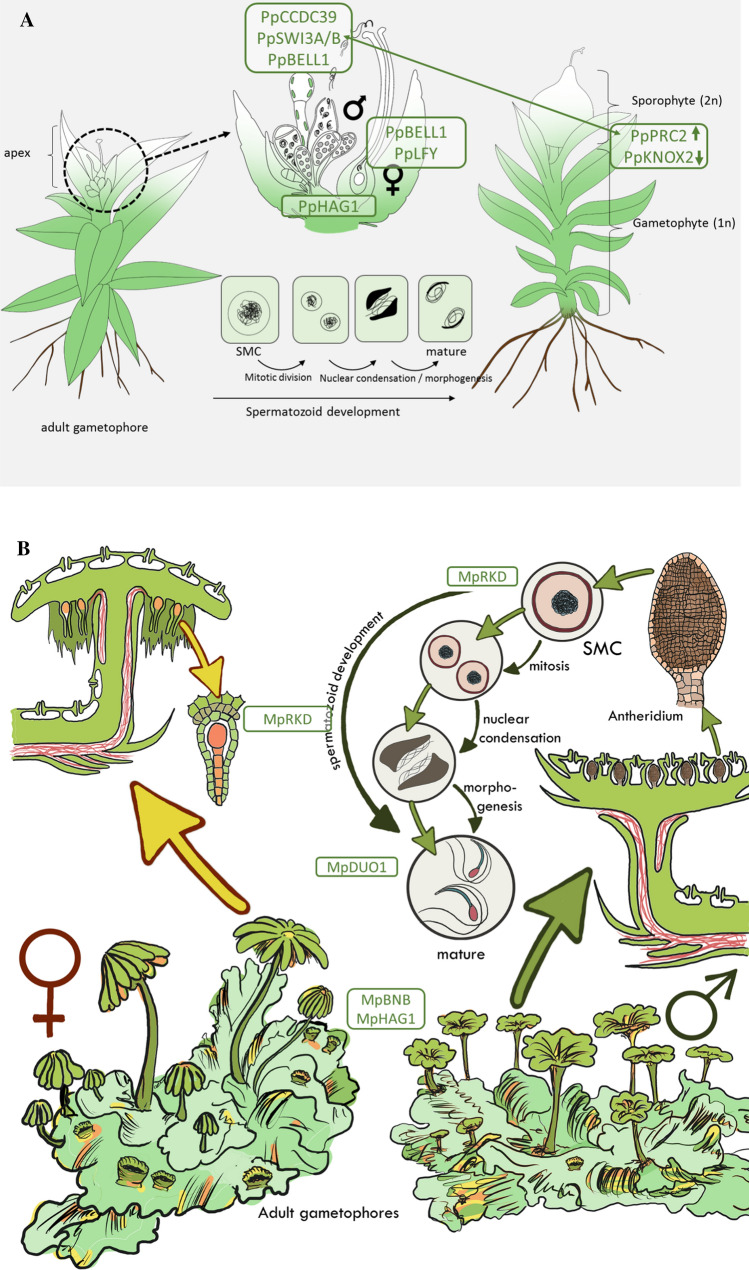
*K*ey regulators of bryophyte sexual reproduction. **a** *P. patens* reproductive organs (gametangia) develop on the apex of each gametophore (leafy shoot); archegonia are the egg-containing female and antheridia the spermatozoid-containing male organs. The latter undergo a complex developmental process starting with the development of the spermatid mother cell (SMC). After mitotic division, each SMC develops into two spermatozoids. During spermatozoid differentiation, autophagy and nuclear condensation take place to finally produce spermatozoids bearing two flagella, two mitochondria and one plastid. Mature antheridia release the motile spermatozoids under wet conditions. The motile male gamete subsequently swims through the archegonial venter to fertilize the egg cell in the cavity. After fertilization, the zygote starts to divide mitotically and embryo development and sporophyte development commences. **b** *M. polymorpha* is dioecious, and there are male and female vegetative thalli from which gametangiophores emerge that will develop gametangia. Reproductive structures and development are principally as described in (**a**), with pegged rhizoids (colored in red) delivering spermatozoids to archegonia (Shimamura 2016). **a, b** Several gene products involved in sexual reproduction (also mentioned in the text) are shown at their site of action, in the following description the function in flowering plants/animals is mentioned in square brackets for comparison. MpBNB [generative cell specification in pollen] (Yamaoka et al. 2018) and MpHAG1 (this study) [reduced fecundity, flower development] are involved in the protrusion of gametangia. Pp*hag1* (this study) leads to impaired gametangia maturation [reduced fecundity, flower development]. PpBELL1 [gynoecium/carpel development], MpRKD [egg cell differentiation] (Rovekamp et al. 2016; Koi et al. 2016) and PpLFY [floral identity] control the female germ line via zygote formation/egg cell maturation/first zygote division (Maizel et al. 2005; Horst et al. 2016b). PpCCDC39 [flagella of male animal sperm] (Meyberg et al. 2020), PpSWI3A/B (this study) [early embryo development, B male germ line; male fertility in animals] and PpBELL1 are involved in male germ line formation through proper flagella formation/late maturation of spermatozoids/putative control of male fecundity (Ortiz-Ramírez et al. 2017). MpRKD [egg cell differentiation] controls the differentiation of antheridial cells into spermatid mother cells, MpDUO1 the last step of spermatozoid formation [spermatid cell formation] (Higo et al. 2018). PpPRC2 controls the haploid body plan (represses the diploid body plan) [reproductive control in plants and animals] (Mosquna et al. 2009; Okano et al. 2009); PpKNOX2 controls the diploid body plan [also in flowering plants] (Sakakibara et al. 2013). PRC2 acts via deposition of H3K27me3 as a silencing mark (Katz et al. 2004; Ikeuchi et al. 2015; Kawashima and Berger 2014), while SWI/SNF acts antagonistically via H3K27 acetylation (shown by double-sided arrow). HAG1 also acts via activating acetylation, of H3K14

Both genes studied here are part of the network that controls the proper orchestration of sexual reproduction. While HAG1 apparently controls the maturation of gametangia, SWI3A/B is involved in the maturation of the spermatozoids (Fig. 14). Loss of function of both genes leads to impairment of male sexual reproduction via nonfertile spermatozoids. On top of that, Pp*hag1* shows a (less pronounced) impairment of female gametangia (archegonia). On the molecular level HAG1 acts via histone 3 (H3) K14 acetylation and the SWI/SNF complex by modulating H3 K27 acetylation (Alver et al. 2017). In that regard, it acts antagonistically to the polycomb repressive complex 2 (PRC2) that is responsible for writing H3K27me3 (Wilson et al. 2010; Pereman et al. 2016). H3K27 has been suggested to serve as an epigenetic toggle in *P. patens* that in particular marks genes important for development (Widiez et al. 2014). Hence, an active SWI/SNF complex probably counteracts PRC2 function, activating genomic regions important for male germ line formation. A similar activating function in terms of gametangia formation might be carried out by HAG1.

## Material and methods

### Candidate gene search

Proteinortho version 5.11 was used for ortholog clustering, options: "-selfblast -identity = 30 -cov = 50 -sim = 0.95" (Lechner et al. 2011) using pre-calculated blastp results obtained via diamond (Buchfink et al. 2015) to derive single copy *P. patens*/*M. polymorpha* orthologs (Fig. S1). The HMM-based TAPscan classification (Wilhelmsson et al. 2017) was applied for assignment of TF/TR families. For the resulting 23 families the orthology status was analyzed via phylogenetic inference. Details of the workflow are shown in Fig. S1. Sequences of the TAP families were obtained by the usage of the web interface TAPScan (https://plantcode.online.uni-marburg.de/tapscan/index.php). All proteins of the respective TAP family for the desired species were extracted and aligned using Mafft L-INS-I v7.310 (Katoh and Standley 2013). After manual trimming of regions represented by a single sequence only, or of questionable alignment quality as determined by Jalview (Waterhouse et al. 2009), the phylogeny was calculated with the help of quicktree-SD (Frickenhaus and Beszteri B: Quicktree-SD, 2008).

The resulting 30 confirmed single copy candidates (Fig. S1) were analyzed via literature research as well as *A. thaliana* TAIR description (https://www.arabidopsis.org/) and *P. patens* GO_annotation (Lang et al. 2018). In total, 19 candidate genes were discarded because there was no information available about the gene in *A. thaliana*, the gene was already tackled in other approaches using bryophytes, or the literature showed no promising phenotype with a strong focus on sexual reproduction. The literature research in many cases allowed to deduce protein function derived from loss-of-function mutants, usually in *A. thaliana*.

For the 11 resulting candidate genes blastp was performed with the respective *A. thaliana* protein sequence against protein sets of selected sequenced genomes (Table S3). The obtained sequences were kept if the alignment was at least 100 positions long and at least 30% identical. Each of the 11 protein sets was aligned using Mafft L-INS-I v7.310 (Katoh and Standley 2013). Additionally, MUSCLE v3.8.31 was used as alignment tool (Edgar 2004). Alignments were manually curated using Jalview 2.8 (Waterhouse et al. 2009), removing identical sequences and trimming as mentioned above. In the case of SWI3 the best suited amino acid substitution model was determined using Prottest 3.4.2 (Darriba et al. 2011) and turned out to be JTT + I + G. Bayesian inference utilizing MrBayes 3.2.7 (Ronquist et al. 2012) was carried out with two hot and cold chains until the average standard deviation of split frequencies was below 0.01 (574,350 generations) and no more trend was observable. In total, 250 trees each were discarded as burn-in. Due to the higher number of sequences, IQ-TREE 1.6.12 (maximum likelihood) was used with automatic model selection for HAG1 (Nguyen et al. 2015). Resulting trees were visualized using FigTree 1.4.3 (http://tree.bio.ed.ac.uk/software/figtree/).

### Localization and expression analyses

For in vivo localization analysis the vector pCABsh_Loc (Hiss et al. 1842) was used. The genes (coding sequence from start to last coding codon, Pp3c2_9560V3.1, Pp3c2_24980V3.1) were fused in frame to GFP (C-terminal GFP tag) under the control of the CABsh Promotor (Hiss et al. 1842) (Fig. S5). Primers Pp3c2_24980#3-EcoR1, Pp3c2_24980#4-Xba1, Pp3c2_9560#4-EcoR1 and Pp3c2_9560#5-Sal1 were used (Table S4).

The candidate genes were analyzed regarding their expression in the species *A. thaliana*, *M. polymorpha* and *P. patens*. Expression data of *M. polymorpha* were reviewed based on data published by (Bowman et al. 2017). *A. thaliana* and *P. patens* expression data were obtained with the help of the eFP Browser (Winter et al. 2007; Schmid et al. 2005; Nakabayashi et al. 2005) (http://bar.utoronto.ca/efp2/Arabidopsis/Arabidopsis_eFPBrowser2.html). *P. patens* expression was analyzed using the webtool PEATmoss (Fernandez-Pozo et al. 2019) (Fig. S4).

### Plant cultivation and phenotypic analyses

*P. patens* ecotype Reute 2017 was cultivated according to Hiss et al. (2017). For sporophyte development, and for crossing analysis, the cultivation was carried out as described in Perroud et al. (2019); long-day (LD) conditions (70 μmol m^−2^ s^−1^, 16-h light, 8-h dark, 22 °C). Gametangia were watered in order to induce sporophyte development at 21 days after short day (SD, 20 µmol m^−2^ s^−1^, 8-h light, 16-h dark, 15 °C) transfer.

#### Selfing and crossing analysis

The number of sporophytes developed 30 days after watering by Reute wildtype as well as loss of function mutants were counted in three independent mutant lines according to Hiss et al. (2017) using a Leica S8Apo binocular (Leica, Wetzlar, Germany). The F1 offspring (sporelings) were counted as described in Hiss et al. (2017) using a fluorescence stereomicroscope SteREO LumarV12 (Carl Zeiss, Oberkochen, Germany). Microsoft Excel 2016 (Microsoft) was used to perform statistical analyses and data visualization. Fisher’s exact test was conducted using https://www.socscistatistics.com/tests/fisher/default2.aspx.

#### Phenotypic analysis of gametangia

A Leica S8Apo binocular (Leica, Wetzlar, Germany) was used for harvesting of sporophytes and gametangia. An upright DM6000 equipped with a DFC295 camera (Leica) was used for taking microscopic pictures. Both devices ran under the control of the Leica Application suite version 4.4. Microsoft PowerPoint was used for processing (brightness and contrast adjustment) the images.

#### Spermatozoid analysis, DAPI staining

Spermatozoid analysis was performed according to Meyberg et al. (2020). Antheridia bundles were harvested 21 days after short-day induction. Spermatozoids were stained with 4′,6-diamidino-2-phenylindole (DAPI). Per sample, antheridia bundles were harvested and opened for spermatozoid release with two ultra-fine forceps (Dumont, Germany) in 7 µl sterile tap water applied to an objective slide. The sample was dried at room temperature (RT) and afterward fixed with 3:1 ethanol/acetic acid for at least five minutes. Samples were finally stained with 0,7 ng/µl DAPI (Roth, Germany) in millipore water. Sealing of samples was performed with nail polish (Marc Cain, Germany). Microscopic images were taken with an upright DM6000 equipped with a DFC295 camera (Leica). Brightness and contrast of microscopy images were adjusted using Microsoft PowerPoint.

### Transmission electron microscopy

Clusters of gametangia were excised and processed for TEM according to Renzaglia et al. (2017). Briefly, specimens were fixed with 2.5% glutaraldehyde in 0.05 M phosphate buffer, pH7.2, washed with buffer, post-fixed with 2% OsO4 and dehydrated in an ascending alcohol preparation using 30, 50, 70, 90 and two times 100% of ethanol. Plants were infiltrated (25% Epon (Fluka® Analytical, USA) in propylene oxide for 2 h; 50% Epon in propylene oxide overnight; next day pure Epon and polymerization. Blocks were thick sections (1.0 mm), stained with toluidine blue and monitored in the light microscope for antheridia at different stages of development. 15–20 blocks each of Reute and mutants were sectioned and observed. Each block contained five to nine antheridia with 7–25 spermatozoids in various planes of section in each antheridium. Over 400 micrographs, representing all stages of development, were collected in the TEM.

### Nucleic acid isolation and RNA-seq

Genomic DNA for genotyping was isolated with a fast extraction protocol, using one plant (approximately 5–10 gametophores) as described in Cove et al. (2009). For RNA-seq 40 apices were harvested in 40 µl RNA later (Qiagen, Hilden, Germany) 21 days after short-day incubation (21 dpi). Plants were watered at 21 dpi to synchronize fertilization. Material was again harvested two days after watering (2 daw = 23 dpi). RNA extraction was performed using the RNeasy plant mini Kit (Qiagen, Hilden, Germany). RNA concentration and quality were measured with the Agilent RNA 6000 Nano Kit using a Bioanalyzer 2100 (Agilent Technologies).

#### Apices RNA-seq and analysis

The MPI genome center Cologne (mpgc.mpipz.mpg.de) performed Truseq RNA-library preparation and RNA-seq Illumina sequencing (HiSeq3000, 18 libraries, 2 × 150 bp, paired end reads). In total, 456,185,664 fragments were sequenced. The analyses were done according to Perroud et al. (2018). DEG calling was done in a strict consensus approach using three R packages (EdgeR, DEseq2, Noiseq). In this approach the intersection of all DEGs called by the three packages was exclusively used for further analyses. The parameters of the three packages were as previously described (Perroud et al. 2018). Downstream analyses, DEG filtering and GO-bias, were performed as in Perroud et al. (2018). The Venn diagrams were calculated with the webtool http://bioinformatics.psb.ugent.be/webtools/Venn/ and Microsoft PowerPoint. Orthology determination was done using the Integrative Orthology Viewer in PLAZA (https://bioinformatics.psb.ugent.be/plaza/versions/plaza_v4_dicots/).

### Vector construction, preparation, moss transfection and genotyping

*P. patens* mutants were obtained performing CRISPR/Cas9 according to Lopez-Obando et al. (2016) with small modifications.

In order to produce loss-of-function mutants, two targets were determined for both genes. Guide RNA (sgRNA) sequences specific to the Pp3c2_9560 and Pp3c2_24980 genes (Fig. S7) were chosen using the webtool CRISPOR 4.97 (Concordet and Haeussler 2018). Expression cassettes sgRNA-Pp3c2_9560#1, sgRNA-Pp3c2_9560#2, sgRNA-Pp3c2_24980#1 and sgRNA-Pp3c2_24980#2, comprising the promoter of the *P. patens* U6 snRNA (Lopez-Obando et al*.* 2016), the 5′-G-N(19)-3′ guide sequences targeting the Pp3c2_9560 or Pp3c2_24980 genes and the tracrRNA scaffold (Table S5) were synthesized by Integrated DNA Technologies (IDT), DE. The different sgRNAs cassettes were cloned in pJet (https://www.thermofisher.com/order/catalog/product/K1231) and pUC19 (https://www.thermofisher.com/order/catalog/product/SD0061#/SD0061) to give psgRNA-Pp3c2_9560#1, psgRNA-Pp3c2_9560#2, psgRNA-Pp3c2_24980#1 and psgRNA-Pp3c2_24980#2. The pAct-Cas9 plasmid used in this study has been described previously (Lopez-Obando et al. 2016).

Moss transfection was performed in the Reute wild-type background according to Hiss et al. (1842). Protoplasts were co-transfected using a mixture (20 µg) of the pAct-Cas9 and psgRNA-Pp3c2_9560#1 and #2 or of the pAct-Cas9 and psgRNA-Pp3c2_24980#1 and #2. Regenerating protoplasts were spread on cellophane disks on basal medium supplemented with 0.33 M Mannitol for 1 week. Plants on cellophane disks were then selected on basal medium supplemented with 25 mg/L G418 (Roth, Germany) to select clones that were transiently transfected. G418-resistant clones were analyzed at the molecular level based on Sanger sequencing of PCR fragments using primers surrounding the targeted locus (Fig. S7). Plants knocked-out for the Pp3c2_9560 gene were identified using primers Pp3c2_9560#1 and Pp3c2_9560#2, and plants knocked-out for the Pp3c2_24980 gene were identified using primers Pp3c2_24980#1 and Pp3c2_24980#2 (Fig. S7). The PCR products were purified using the Hi Yield® Gel/PCR DNA Fragment Extraction Kit (Süd-Laborbedarf-Gauting) and sequenced at Macrogen Europe B.V. The resulting sequences were analyzed using Sequencher 5.1 (https://www.genecodes.com/). Consequences, at the protein level, of the CRISPR-Cas mediated mutations on the SWI3A/B and HAG1 genes are shown in Fig. S7. PCR primers used in this study are listed in Table S4.

### Liverwort culture conditions and transfection

The liverwort studies were performed in the *M. polymorpha ssp. ruderalis,* ecotype BoGa background, which was kindly provided by Sabine Zachgo (University of Osnabrück). The sterile cultivation and the induction of the reproductive life cycle stages were carried out according to Althoff et al. (2014). *M. polymorpha* mutants were obtained by performing a double CRISPR/Cas9 approach according to Sugano et al. (2018; Ishizaki et al. 2015). The targets (gRNA 20 bp) were determined with the help of the webtool CRISPOR 4.97 (Concordet and Haeussler 2018). For every loss-of-function mutant, two targets were determined. Targets and primers are shown in Table S4/S5. The first target was designed next to the start codon, the second target next to the stop codon to obtain a deletion of the gene Mapoly0187s0003.1. The determined target was annealed as double strand and ligated in the vector already equipped with the sgRNA backbone. The targets were ordered as forward and reverse primers with BsaI overhangs. Genotyping results for Mp*hag1* are shown in Fig. S16. The pMpGE_EN03 was used as entry vector (Sugano et al. 2018). The first sgRNA next to the start codon of the respective gene (sgRNA1) was always cloned in pMpGE010 equipped with the Cas9. The second sgRNA next to the stop codon of the respective gene (sgRNA2) was always cloned in pMpGWB401 (Sugano et al. 2018; Ishizaki et al. 2015). *Agrobacterium*‐mediated sporeling transformation was carried out using both gRNA‐containing vectors. The transgenic lines were selected using 100 μg * ml^−1^ cefotaxime, 10 μg * ml^−1^ hygromycin, 5 μg * ml^−1^ G418 and genotypting. The obtained male and female lines were analyzed using primers surrounding the targeted locus (Fig. S6). First, absence of the wild-type locus was tested using primers Mapoly0187s0003#1 and Mapoly0187s0003#3 and then the mutant locus was analyzed using primers Mapoly0187s0003#1 and Mapoly0187s0003#2 (Fig. S16).

#### Author contribution statement

ACG, NFP and SAR performed the phylogenetic analyses with help by PKIW and KKU; ACG, FN and CG planned and generated the mutants; ACG and ZL analyzed the mutants; ACG generated the RNA-seq data; ACG and FBH analyzed it; ACG, NFP and ZL carried out expression analyses; ACG, RM, ZL and MS generated figures; KSR performed the TEM studies with help by ACG and RM; ACG and SAR wrote the paper with help by all authors; SAR conceived of and supervised the project with contributions by CG and RM.

## Supplementary Information

Below is the link to the electronic supplementary material.

Supplementary file1 (DOCX 21831 kb)

Supplementary file2 (XLSX 10991 kb)

## Data Availability

RNA-seq data from this study are available at the NCBI SRA under PRJNA684003. Reviewer access: https://dataview.ncbi.nlm.nih.gov/object/PRJNA684003?reviewer=k800vrlv58aiq8d7aohd751h97.
