# Supplementary material for: HAG1 and SWI3A/B control of male germ line development in P. patens suggests conservation of epigenetic reproductive control across land plants
Source: Plant Reprod. 2021 Apr 11;34(2):149–73. doi: 10.1007/s00497-021-00409-0 (PMC8128824; doi:10.1007/s00497-021-00409-0)
Supplement: Supplementary file 1 — Supplementary file1 (DOCX 21831 kb) [file 497_2021_409_MOESM1_ESM.docx]

**Supplementary material for Genau et al.´**


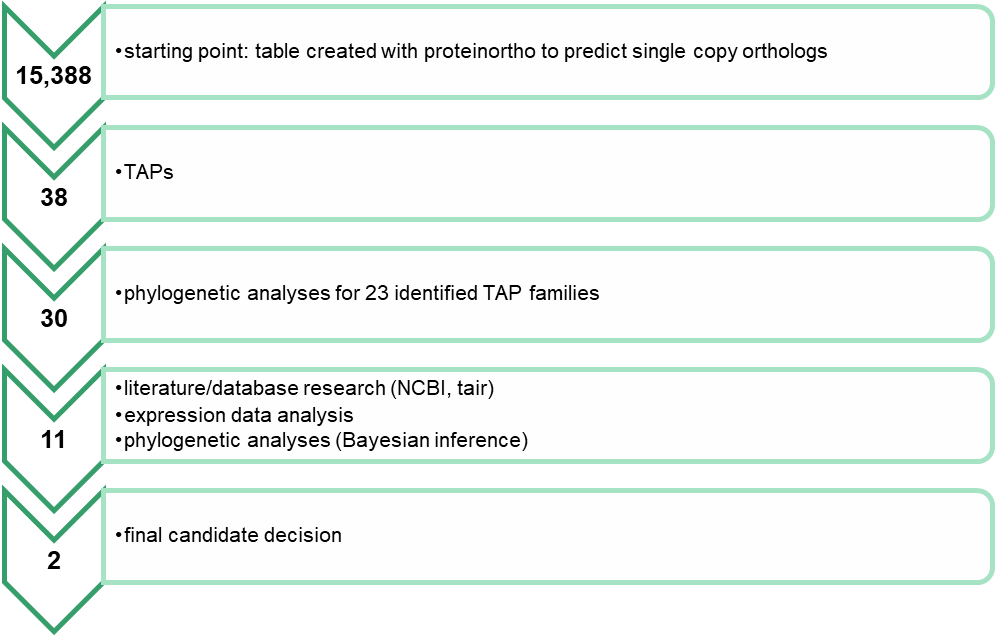


**Figure S1: Workflow of phylogenetic singly copy TF/TR screening.**

Based on a proteinortho [1] run 15,388 *P. patens,* 12,478 *M. polymorpha* and 14755 *A. thaliana* orthologous groups were filtered according to single copy status in each species. These proteins were scanned further using TAPscan [2], yielding 38 proteins classified into 23 TAP (TF or TR) families (GNAT, MYB-related, SET/Sigma70-like, FHA, PcG_MSI, Jumonji_Other, IWS1, C3H, C2H2, bHLH, TANGO2, tify, TUB, WRKY, MADS, Med7, O-FucT, BES1, CCAAT_HAP3, GRAS, HMG, ARID and Med6). For these 23 families, phylogenetic inference using Neighbour-Joining with quicktree-SD [3] was performed using the proteins of *Arabidopsis thaliana*, *Anthoceros agrestis*, *Marchantia polymorpha*, *Physcomitrium patens* as well as *Oryza sativa* to verify single copy status in the three bryophytes of interest, leading to removal of 8 genes on account of unclear/lack of orthology. For the remaining 30 proteins initial literature and expression analyses were conducted, narrowing them down to 11 candidates (Table S1) from which 2 were selected for this study.


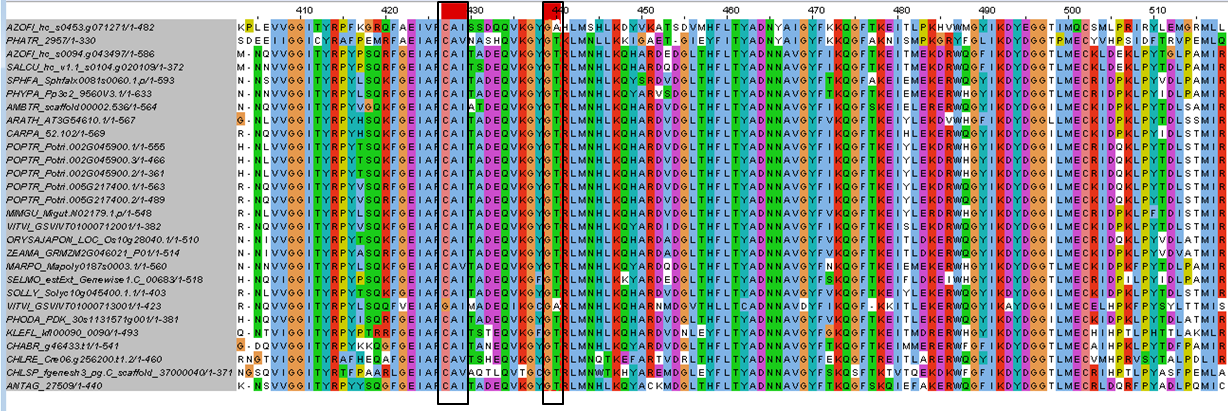
**Figure S2*:* Multiple sequence alignment of the GNAT domain of HAG1.**

The conserved Coenzyme A binding pocket is marked by black boxes.


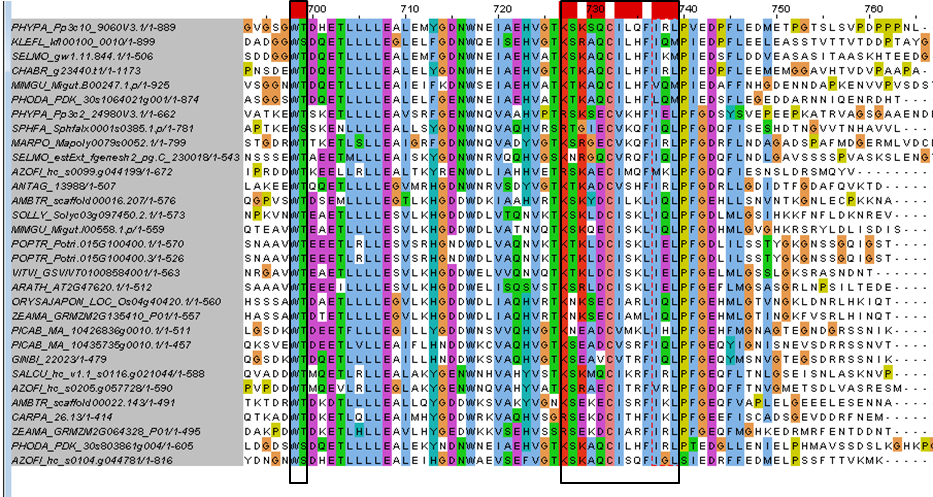
**Figure S3: Multiple sequence alignment of part of the SANT/MYB domain of SWI3A/B.**

The potential DNA binding sites of the MYB domain are marked by black boxes.


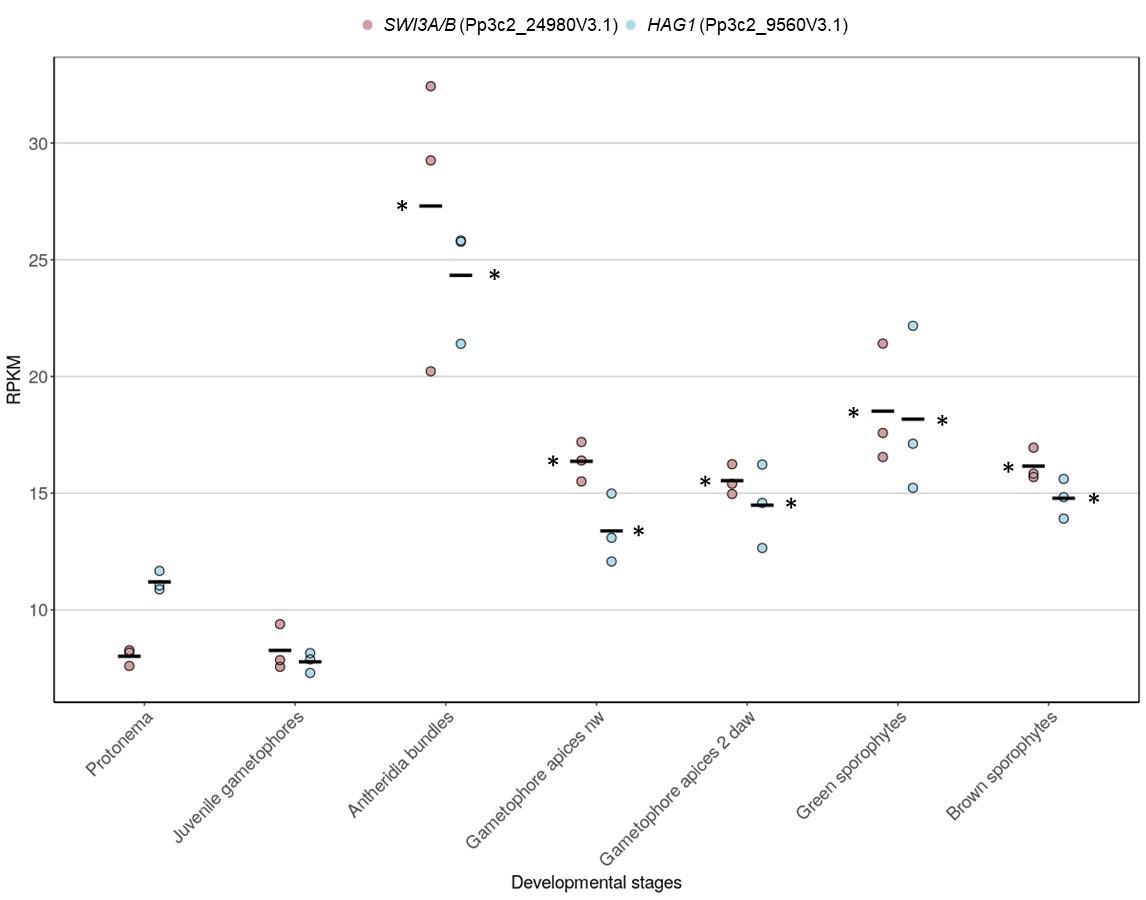


**Figure S4: RNA-seq expression profile of Pp*HAG1* (Pp3c2_9560V3.1, histone acetyltransferase) and Pp*SWI3A/B* (Pp3c2_24980V3.1, chromatin remodelling complex subunit).**

Data are from [4], antheridia bundles from [5] and apices from this study. The highest expression of both genes can be detected in antheridia bundles, but expression in all reproductive stages shown is significantly (t-test, p < 0.05; asterisks) higher than in the vegetative control (protonema and juvenile = asexual gametophores). Expression data in archegonia, representing the female germ line [6] is not significantly higher than in vegetative tissue (data not shown because from microarray / different scale). Gametophore apices feature gametangia (female archegonia and male antheridia). Green sporophytes are pre-meiotic, brown sporophytes post-meiotic. RPKM is Reads Per Kbp (of transcript) and Million (of reads); data were generated according to standardized procedures allowing to compare across samples [7].


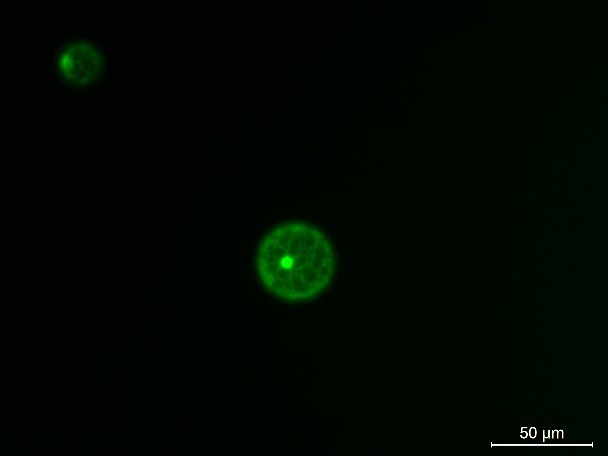

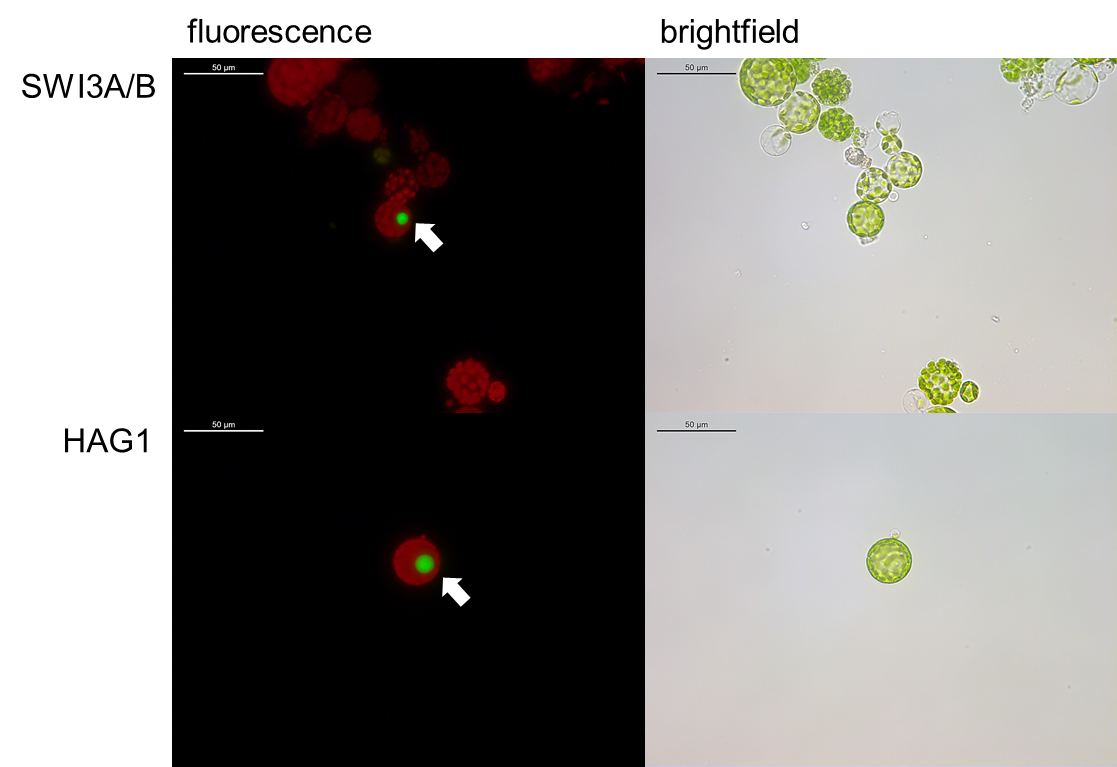


**Figure S5: *In vivo* localization analysis.**

Transient expression of PpSWI3A/B and PpHAG1 fused to a C-terminal GFP. The white arrows indicate the nuclear localization. A protoplast transiently expressing GFP in the nucleo-/cytoplasm is shown for comparison.

200 bp

***Pp3c2_9560***

(5806 bp)

ATG

Stop

sgRNA1

sgRNA2

**#2**

**#1**

200 bp

***Pp3c2_24980***

(5562 bp)

Stop

sgRNA1

sgRNA2

**#2**

ATG

**#1**

400 bp

***Mapoly0187s0003***

(9065 bp)

Stop

sgRNA1

sgRNA2

**#2**

ATG

**#1**

**# 3**

**Figure S6: Structure of the Pp3c2_9560, Pp3c2_24980 and Mapoly0187s0003 targeted genes.**

Structure of the *Pp3c2_9560, Pp3c2_24980* and *Mapoly0187s0003* genes with their respective sgRNAs positions. Boxes represent the exons and black lines represent the introns. The two sgRNAs positions are indicated (in red). Green arrows represent the primers used for PCR and sequencing.

A

1 100

Wild-Type TGCTTCCAAGGAGGCGAAACGTAATGCGTGCGAGGTGGGGTTCTTTGGAATGAGGAGCAGGAGTGAGGTGTGAATTCTGGTAGTTTATGGGGAGTCTTTG

*Swi3a/b-1* TGCTTCCAAGGAGGCGAAACGTAATGCGTGCGAGGTGGGGTTCTTTGGAATGAGGAGCAGGAGTGAGGTGTGAATTCTGGTAGTTTATGGGGAGTCTTTG

*Swi3a/b-2* TGCTTCCAAGGAGGCGAAACGTAATGCGTGCGAGGTGGGGTTCTTTGGAATGAGGAGCAGGAGTGAGGTGTGAATTCTGGTAGTTTATGGGGAGTCTTTG

*Swi3a/b-3* TGCTTCCAAGGAGGCGAAACGTAATGCGTGCGAGGTGGGGTTCTTTGGAATGAGGAGCAGGAGTGAGGTGTGAATTCTGGTAGTTTATGGGGAGTCTTTG

101 200

Wild-Type TTGGGGGGAAGCTGGGAGTTGATGATTGGCAGGGTGGAGGTACAGTGGGTTTGTTCGAAGGTGATGTGTATTAGAAAGGGGAGGGAGTGAGCGTGGATCA

*Swi3a/b-1* TTGGGGGGAAGCTGGGAGTTGATGATTGGCAGGGTGGAGGTACAGTGGGTTTGTTCGAAGGTGATGTGTATTAGAAAGGGGAGGGAGTGAGCGTGGATCA

*Swi3a/b-2* TTGGGGGGAAGCTGGGAGTTGATGATTGGCAGGGTGGAGGTACAGTGGGTTTGTTCGAAGGTGATGTGTATTAGAAAGGGGAGGGAGTGAGCGTGGATCA

*Swi3a/b-3* TTGGGGGGAAGCTGGGAGTTGATGATTGGCAGGGTGGAGGTACAGTGGGTTTGTTCGAAGGTGATGTGTATTAGAAAGGGGAGGGAGTGAGCGTGGATCA

201 300

Wild-Type GAGGAGGAGAGGTAGACATGGTGAACCCGGCGAGCGTGCCTACGGGGTTGTCCAAAGTGAGGAGGGAGAGGGAAGAGGAGAACGCGGGTGTGGATGTTCC

*Swi3a/b-1* GAGGAGGAGAGGTAGACATGGTGAACCCGGCGAGCGTGCCTACGGGGTTGTCCAAAGTGAGGAGGGAGAGGGAAGAGGAGAACGCGGGTGTGGATGTTCC

*Swi3a/b-2* GAGGAGGAGAGGTAGACATGGTGAACCCGGCGAGCGTGCCTACGGGGTTGTCCAAAGTGAGGAGGGAGAGGGAAGAGGAGAACGCGGGTGTGGATGTTCC

*Swi3a/b-3* GAGGAGGAGAGGTAGACATGGTGAACCCGGCGAGCGTGCCTACGGGGTTGTCCAAAGTGAGGAGGGAGAGGGAAGAGGAGAACGCGGGTGTGGATGTTCC

301 400

Wild-Type GAGCAAGAAGAGCAGGAAAGCTGGGCAGAGCGGAGTGCCGGAGCCCACTCCCGAAG**CGG**GGAATCAAATTGCGTCGGGGAAAGGAGAGGATGAAAACAAT

*Swi3a/b-1* GAGCAAGAAGAGCAGGAAAGCTGGGCAGAGCGGAGTGCCGGAGCCCACT...................................................

*Swi3a/b-2* GAGCAAGAAGAGCAGGAAAGCTGGGCAGAGCGGAGTGCCGGAGCCCACT...................................................

*Swi3a/b-3* GAGCAAGAAGAGCAGGAAAGCTGGGCAGAGCGGAGTGCCGGA..........................................................

401 500

Wild-Type GGAACGTTGGAAGAGAATGCAGAGGGGGCTCCCCAAGAAAATGCAGAAATTAGTCACAAGAGTGACCCAGCTTCCGTGGAACCATCAGCTATACCCACAG

*Swi3a/b-1* ....................................................................................................

*Swi3a/b-2* ....................................................................................................

*Swi3a/b-3* ....................................................................................................

501 600

Wild-Type CCACTCCAGAGGCTGCGACC**CGG**CCTGAAGCGCCTACTCAAGAGGTGTACAGAATACCCAGCTATGCAGGTGCTTGAAACTCAGAATGCCTTAGTATTCC

*Swi3a/b-1* .......................CCTGAAGCGCCTACTCAAGAGGTGTACAGAATACCCAGCTATGCAGGTGCTTGAAACTCAGAATGCCTTAGTATTCC

*Swi3a/b-2* ..................CCCGGCCTGAAGCGCCTACTCAAGAGGTGTACAGAATACCCAGCTATGCAGGTGCTTGAAACTCAGAATGCCTTAGTATTCC

*Swi3a/b-3* ............................AGCGCCTACTCAAGAGGTGTACAGAATACCCAGCTATGCAGGTGCTTGAAACTCAGAATGCCTTAGTATTCC

601 700

Wild-Type GGTGTTGTACTGGAGAATAGGGAACTGCTTGATGTATGTGTGCGTGTGTATGTGTGGATAGGCGTAGGTTTCTCTAGCTTGTTCTAATATTGTGCGCATG

*Swi3a/b-1* GGTGTTGTACTGGAGAATAGGGAACTGCTTGATGTATGTGTGCGTGTGTATGTGTGGATAGGCGTAGGTTTCTCTAGCTTGTTCTAATATTGTGCGCATG

*Swi3a/b-2* GGTGTTGTACTGGAGAATAGGGAACTGCTTGATGTATGTGTGCGTGTGTATGTGTGGATAGGCGTAGGTTTCTCTAGCTTGTTCTAATATTGTGCGCATG

*Swi3a/b-3* GGTGTTGTACTGGAGAATAGGGAACTGCTTGATGTATGTGTGCGTGTGTATGTGTGGATAGGCGTAGGTTTCTCTAGCTTGTTCTAATATTGTGCGCATG

701 800

Wild-Type AAAATATGGAGACTTGGAGGTTTGTTTTGGAGGTTTGTGATATGCATGATTTAAAAAATAAATAAATAAATAAATAAAATCAATTCATTAACTGGTGCAA

*Swi3a/b-1* AAAATATGGAGACTTGGAGGTTTGTTTTGGAGGTTTGTGATATGCATGATTTAAAAAATAAATAAATAAATAAATAAAATCAATTCATTAACTGGTGCAA

*Swi3a/b-2* AAAATATGGAGACTTGGAGGTTTGTTTTGGAGGTTTGTGATATGCATGATTTAAAAAATAAATAAATAAATAAATAAAATCAATTCATTAACTGGTGCAA

*Swi3a/b-3* AAAATATGGAGACTTGGAGGTTTGTCTTGGAGGTTTGTGATATGCATGATTTAAAAAATAAATAAATAAATAAATAAAATCAATTCATTAACTGGTGCAA

801 900

Wild-Type TTACGTTGTTTCCTAAGCTATCTGCCAAGGCAGTCTGCCAGTTGTGCACTTTGTTTGATCGGAGTCTGAGAAACTTTGTGAATATCTTGAACCTGGAGTG

*Swi3a/b-1* TTACGTTGTTTCCTAAGCTATCTGCCAAGGCAGTCTGCCAGTTGTGCACTTTGTTTGATCGGAGTCTGAGAAACTTTGTGAATATCTTGAACCTGGAGTG

*Swi3a/b-2* TTACGTTGTTTCCTAAGCTATCTGCCAAGGCAGTCTGCCAGTTGTGCACTTTGTTTGATCGGAGTCTGAGAAACTTTGTGAATATCTTGAACCTGGAGTG

*Swi3a/b-3* TTACGTTGTTTCCTAAGCTATCTGCCAAGGCAGTCTGCCAGTTGTGCACTTTGTTTGATCGGAGTCTGAGAAACTTTGTGAATATCTTGAACCTGGAGTG

901 915

Wild-Type ATGGAGTTGCATAGT

*Swi3a/b-1* ATGGAGTTGCATAGT

*Swi3a/b-2* ATGGAGTTGCATAGT

*Swi3a/b-3* ATGGAGTTGCATAGT

B

1 100

Wild-Type TCCAACGGTGATTCTCGCAGCGTAAGAATCAATCACTGCTCTAGATTGAAATGGTGGCAGAGATGGCGTCACAGCAGTTCTCGGTGACGCTGTCGCCATC

*hag1-1* TCCAACGGTGATTCTCGCAGCGTAAGAATCAATCACTGCTCTAGATTGAAATGGTGGCAGAGATGGCGTCACAGCAGTTCTCGGTGACGCTGTCGCCATC

*hag1-2* TCCAACGGTGATTCTCGCAGCGTAAGAATCAATCACTGCTCTAGATTGAAATGGTGGCAGAGATGGCGTCACAGCAGTTCTCGGTGACGCTGTCGCCATC

*hag1-3* TCCAACGGTGATTCTCGCAGCGTAAGAATCAATCACTGCTCTAGATTGAAATGGTGGCAGAGATGGCGTCACAGCAGTTCTCGGTGACGCTGTCGCCATC

101 200

Wild-Type GCCATCGCCATCGCCGTCGTATTCGTCATTAAGCGGGTCCAGTCAGAAACGGAAACGATCCGCGCAGGATGCTGCTGCAACGGCAGACGAGTTTGTGGCA

*hag1-1* GCCATCGCCATCGCCGTCGTATTCGTCATTAAGCGGGTCCAGTCAGAAACGGAAACGATCCGCGCAGGATGCTGCTGCAACGGCAGACGAGTTTGTGGCA

*hag1-2* GCCATCGCCATCGCCGTCGTATTCGTCATTAAGCGGGTCCAGTCAGAAACGGAAACGATCCGCGCAGGATGCTGCTGCAACGGCAGACGAGTTTGTGGCA

*hag1-3* GCCATCGCCATCGCCGTCGTATTCGTCATTAAGCGGGTCCAGTCAGAAACGGAAACGATCCGCGCAGGATGCTGCTGCAACGGCAGACGAGTTTGTGGCA

201 300

Wild-Type AACTCCCCGAACCCCTTCCTCCAACCACACTCCAACGCTCACATCAAAGCGTCCACCAATCTTCTCAACGG**TGG**TGACCCTGTCGGCTCCCTCAACCAGC

*hag1-1* AACTCCCCGAACCCCTTCCTCCAACCACACTCCAACGCTCACATCAAAGCGTCCACCAATCTTCTCAC................................

*hag1-2* AACTCCCCGAACCCCTTCCTCCAACCACACTCCAACGCTCACATCAAAGCGTCCACCAATCTAAACCCTAAACCTAAACCCTAAAC..............

*hag1-3* AACTCCCCGAACCCCTTCCTCCAACCACACTCCAACGCTCACATCAAAGCGTCCACCAATCTTCTCAATC..............................

301 400

Wild-Type AGCAGCATCCGATTATAATGCCGTTGTCCTCGCTGGACTCGCAGCACACGCTGCCGGAAAATCCCGGGAGCTCGCTGGAGGATGAGGACGATGGGGAAAA

*hag1-1* ....................................................................................................

*hag1-2* .................................................................................................... *hag1-3* ....................................................................................................

401 500

Wild-Type TGGAGGGCATCATCACGTCATGTCGTCCGATGGGGTCAAGAATGGGCCTAGCTATGGTCGGTTCGAAGGTGCGAACGG**CGG**TGGGGATGGGGATGAAGAG

*hag1-1* ................................................................................GTGGGGATGGGGATGAAGAG

*hag1-2* ...........................................................................CGGCGGTGGGGATGGGGATGAAGAG

*hag1-3* ...........................................................................CGGCGGTGGGGATGGGGATGAAGAG

501 600

Wild-Type GAGGACGACGATGAGGAGGAAGGCGAAGCGAACGAAGAAGAAGGAGAGCACGACGAAGGTAAAACCCTAAACCTTGGTATGCCTTCGCAATCAGCGTTCT

*hag1-1* GAGGACGACGATGAGGAGGAAGGCGAAGCGAACGAAGAAGAAGGAGAGCACGACGAAGGTAAAACCCTAAACCTTGGTATGCCTTCGCAATCAGCGTTCT

*hag1-2* GAGGACGACGATGAGGAGGAAGGCGAAGCGAACGAAGAAGAAGGAGAGCACGACGAAGGTAAAACCCTAAACCTTGGTATGCCTTCGCAATCAGCGTTCT

*hag1-3* GAGGACGACGATGAGGAGGAAGGCGAAGCGAACGAAGAAGAAGGAGAGCACGACGAAGGTAAAACCCTAAACCTTGGTATGCCTTCGCAATCAGCGTTCT

601 700

Wild-Type TGCTCAGGCATTGGAACGGATCCTGACGATTGTGATCTGTTGTCTTATTCTTTCTTTCTTTCTTTATTTTATTTTTAAAGCCTACTTTGCAACTTGAAGA

*hag1-1* TGCTCAGGCATTGGAACGGATCCTGACGATTGTGATCTGTTGTCTTATTCTTTCTTTCTTTCTTTATTTTATTTTTAAAGCCTACTTTGCAACTTGAAGA

*hag1-2* TGCTCAGGCATTGGAACGGATCCTGACGATTGTGATCTGTTGTCTTATTCTTTCTTTCTTTCTTTATTTTATTTTTAAAGCCTACTTTGCAACTTGAAGA

*hag1-3* TGCTCAGGCATTGGAACGGATCCTGACGATTGTGATCTGTTGTCTTATTCTTTCTTTCTTTCTTTATTTTATTTTTAAAGCCTACTTTGCAACTTGAAGA

701 800

Wild-Type CTATGCAGGAGCTTTAGCAGAGGGCTGTTTGGAATGTTGGATAATGATCTTGCTTGTTTTAACTTTTAAGATGACGGTAGTGTTTGGCACAATTGATCGT

*hag1-1* CTATGCAGGAGCTTTAGCAGAGGGCTGTTTGGAATGTTGGATAATGATCTTGCTTGTTTTAACTTTTAAGATGACGGTAGTGTTTGGCACAATTGATCGT

*hag1-2* CTATGCAGGAGCTTTAGCAGAGGGCTGTTTGGAATGTTGGATAATGATCTTGCTTGTTTTAACTTTTAAGATGACGGTAGTGTTTGGCACAATTGATCGT

*hag1-3* CTATGCAGGAGCTTTAGCAGAGGGCTGTTTGGAATGTTGGATAATGATCTTGCTTGTTTTAACTTTTAAGATGACGGTAGTGTTTGGCACAATTGATCGT

801 880

Wild-Type ATCTTTTTATTTTTGTTTTTTTACGGCTGCCTTAATTTGTGGAAACTGACTGAGAATGTGATCGTATCTCGGGAGCACCT

*hag1-1* ATCTTTTTATTTTTGTTTTTTTACGGCTGCCTTAATTTGTGGAAACTGACTGAGAATGTGATCGTATCTCGGGAGCACCT

*hag1-2* ATCTTTTTATTTTTGTTTTTTTACGGCTGCCTTAATTTGTGGAAACTGACTGAGAATGTGATCGTATCTCGGGAGCACCT

*hag1-3* ATCTTTTTATTTTTGTTTTTTTACGGCTGCCTTAATTTGTGGAAACTGACTGAGAATGTGATCGTATCTCGGGAGCACCT

**Figure S7: Sequence aligment of the targeted regions in the *HAG1* and *SWI3A/B* genes in the wild-type and respective mutants.**

Alignment of (A) *SWI3A/B* and *swi3a/b-1*, *swi3a/b-2* and *swi3a/b-3* CRISPR-Cas targeted region, and (B) *HAG1* and *hag1-1*, *hag1-2* and *hag1-3* CRISPR-Cas targeted region. *SWI3A/B* (Pp3c2_24980); *HAG1* (Pp3c2_9560). Sequences of the sgRNAs used in this study to produce the mutants are in green (PAMs in bold). Substitutions, deletions, or insertions are indicated in red. Primers used to amplify and sequence the *HAG1* or *SWI3A/B* regions are in blue.

**
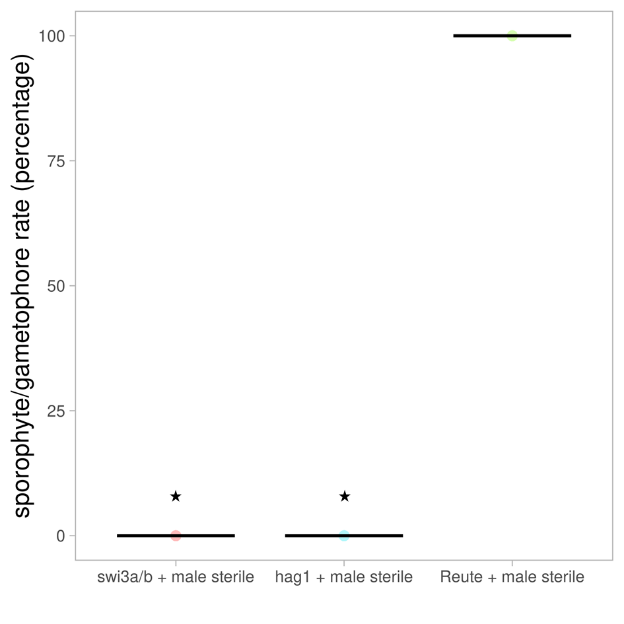
**

**Figure S8: Crossing with a male infertile strain to test for male impairment.**

Pp*hag1* (blue), Pp*swi3a/b* (red) and the control (Reute, green) were crossed with a male infertile strain [5]. Reute developed 100% of sporophytes per gametophore (through selfing), whereas Pp*swi3a/b* and Pp*hag1* developed no sporophytes, which is a significant reduction (asterisks) (p < 0,01, Fisher’s exact test). The total number of gametophores analysed per mutant/control was 73 each. The dots indicate the rate of sporophytes per gametophore as percentage relative to the total number of gametophores.


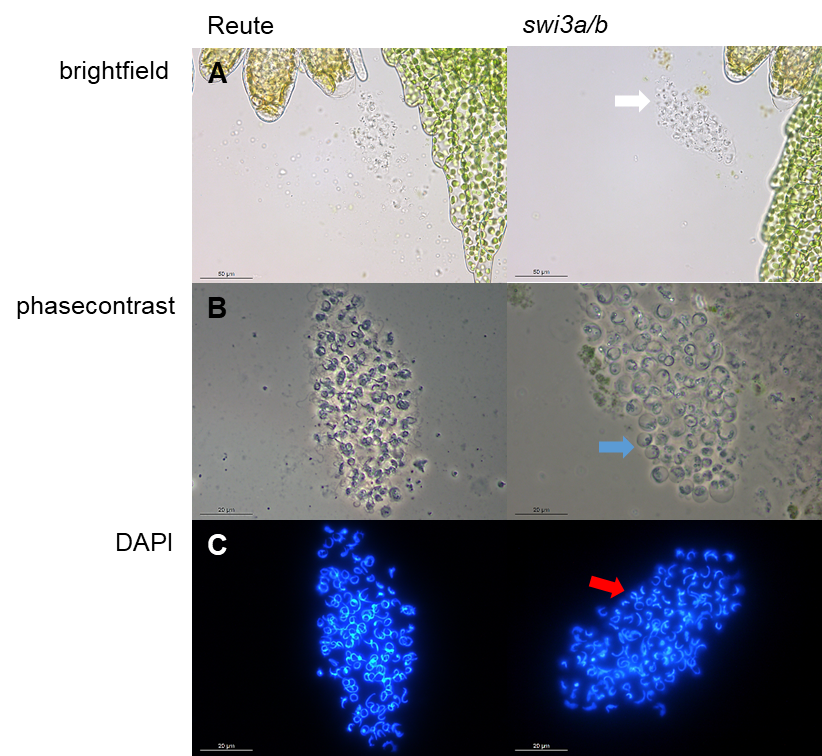


**Figure S9: Spermatozoid analysis of Pp*swi3a/b* 21 dpi.**

A) While spermatozoids of Reute as control start to move and swim shortly after release, *swi3a/b* spermatozoids are unable to swim and stick together without moving away from the antheridial bundle (indicated by white arrow). B) Phase contrast images of Reute and Pp*swi3a/b*. The spermatozoids of *swi3a/b* show caviar-like structures (indicated by blue arrow); cf. Figs. 6/S11. C) The DAPI staining shows fully condensed nuclei in Reute, whereas Pp*swi3a/b* nuclei show slight structural abnormalities / show (indicated by red arrow, *cf.* Fig. 6).

**
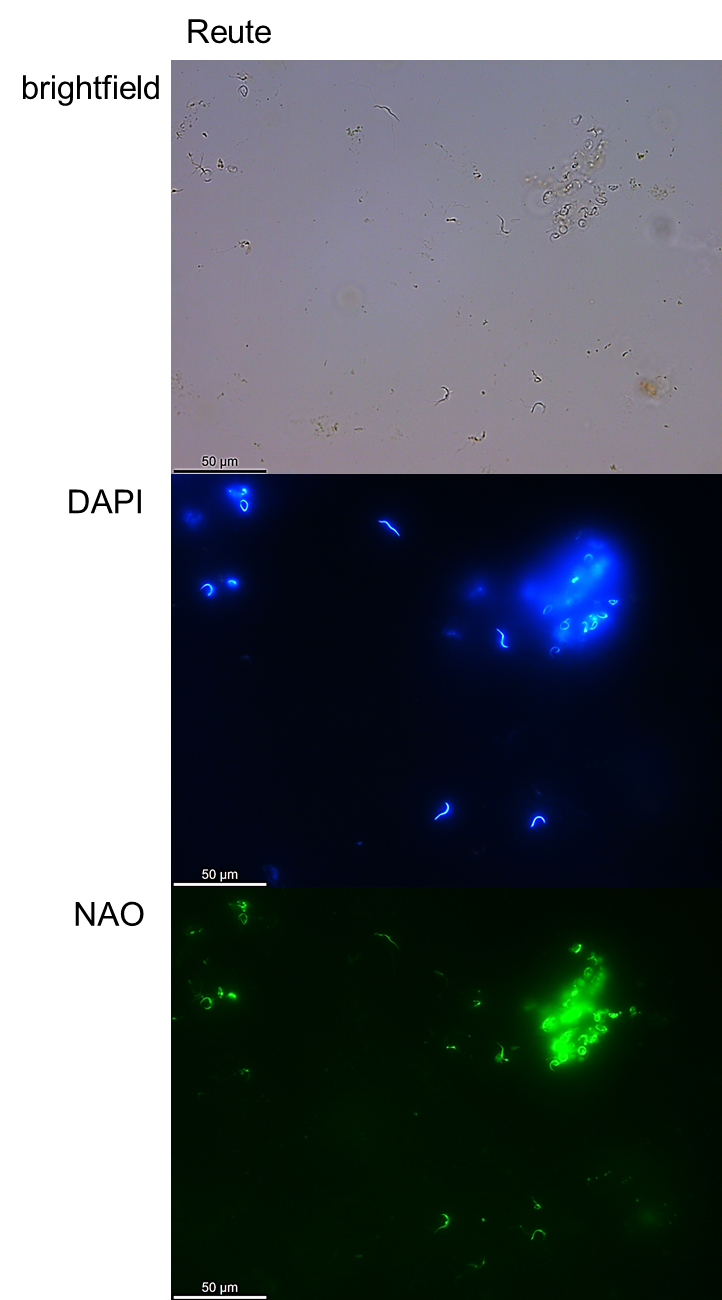
**

**Figure S10: Spermatozoid analysis via a double staining with DAPI and NAO.**

Several released Reute (control) spermatozoids are shown with their slender shape and fully reduced cytoplasm.

**
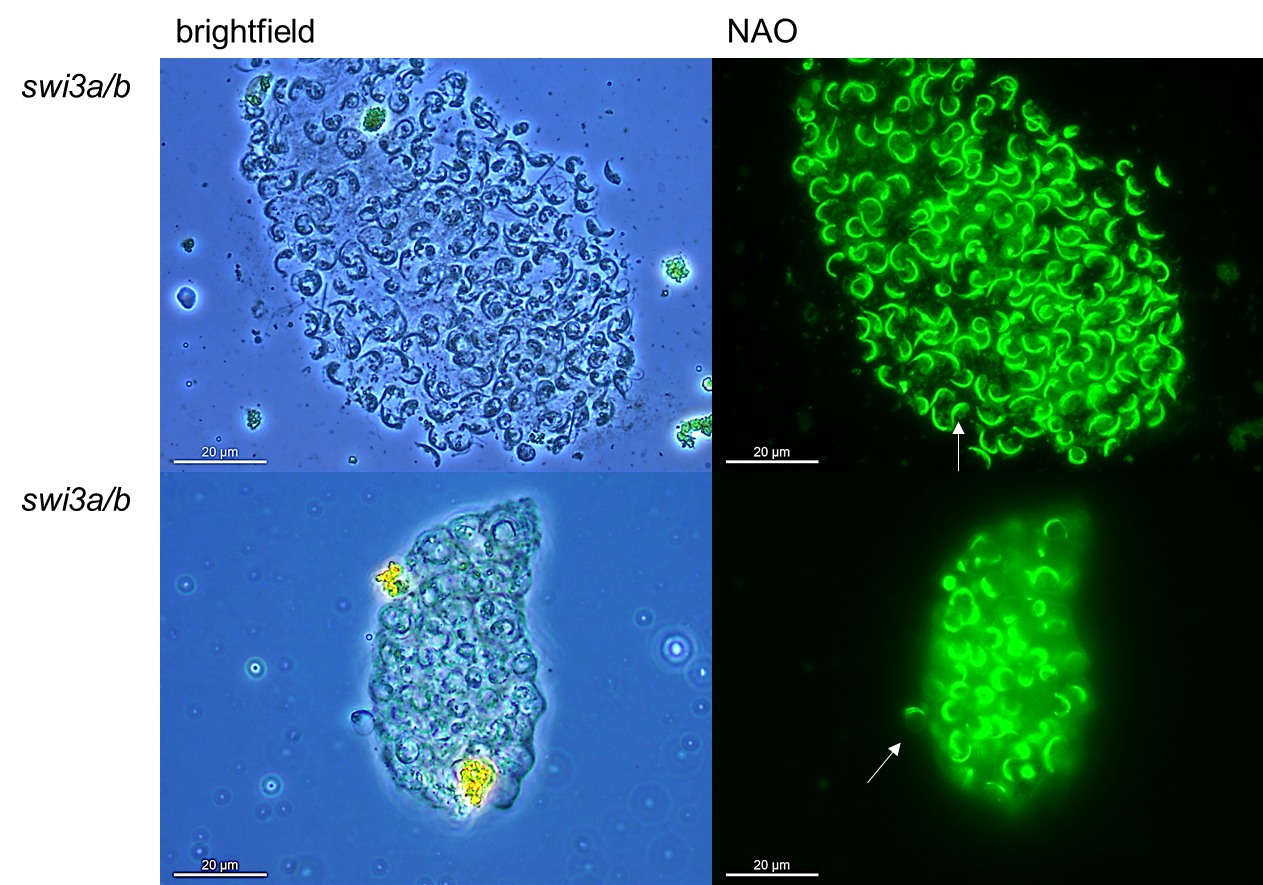
**

**Figure S11: Spermatozoid analysis via staining with NAO.**

Ppswi3a/b antheridia release spermatozoids, which however are embedded in a matrix that may form a caviar-like bubbles (in which the condensing nucleus is surrounded by the extraplasmatic matrix in which gametes differentiate, bottom panel), and/or show incomplete cytoplasmic reduction (upper panel) (white arrows).


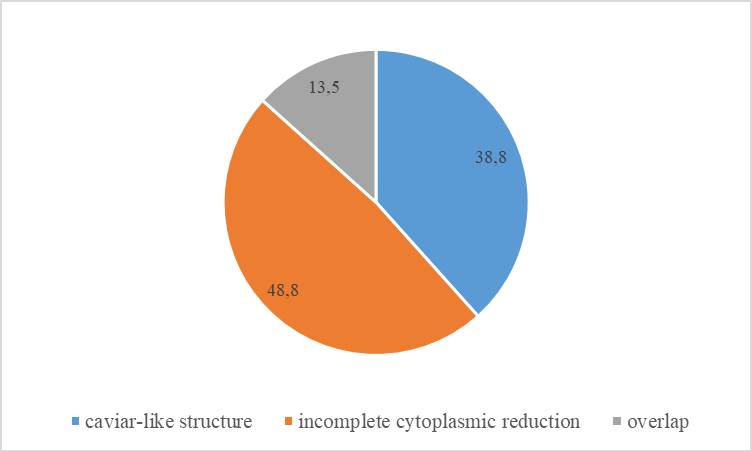


**Figure S12: Light microscopical analysis of Pp*swi3a/b* antheridia.**

The Caviar-like structure could be seen in 37.8% of the analyzed pictures, whereas the incomplete cytoplasmic reduction was observed in 48.8% of analyzed pictures. An overlap of caviar-like structures and incomplete cytoplasmic reduction was observed in 13.5% of analyzed pictures. 37 pictures were analyzed in total.

**Figure S13: Antheridial develop in Reute and *Ppswi3a/b***

Antheridial development and early spermatids before origin of locomotory apparatus are identical in Reute control and Pp*swi3a/b*. A) Light micrograph (LM) longitudinal section of a developing antheridium with stalk cells (s) showing triangular apical cell (ac) and beginning of biseriate filament (bf) next to a mucilage hair (mh) (Reute). B) LM cross section of the body of an antheridium with two primary androgones (a) surrounded by newly formed jack cell (j) (Pp*swi3a/b*). C) LM longitudinal section of developing antheridium with spermatogenous tissue (st) surrounded by jacket cells (j) (Reute). D) Transmission electron micrograph (TEM) of dense spermatogenous cell full of ribosomes and endoplasmic reticulum with large nucleus (n), mitochondria (m) and two plastids (p) at the poles preparing for mitosis. Plasmodesmata (pd) are abundant in young walls (Reute). E) TEM of nascent spermatid with large nucleus (n) and dense cytoplasm containing abundant ribosomes, ER, vesicles (v), a single plastid (p) and numerous plasmodesmata (pd), surrounded by jacket cells (j) (Pp*swi3a/b*). F) Young spermatids deposit thick cell wall (*) via vesicles (v) and round out prior to developing the locomotory apparatus. Pairs of spermatids with large nuclei (n) are connected by expanded plasmodesmata that form cytoplasmic bridges (cb). Jacket cells (j) border the antheridium (Pp*swi3a/b*). Bars: A-C = 10 µm; D-F = 2mm.

**Figure S14: TEM details of mature, unreleased spermatozoids in Reute and Pp*swi3a/b***

Reute control in left column and Pp*swi3a/b* mutant in right column. A) Cross section of front of the spermatozoid showing condensed nucleus (n) and mitochondrion (m) under a broad spline (sp) composed of a band of 23 microtubules. The 9+2 flagellum (f) and microtubules (mt) that identify the insertion of the second flagellum are visible. The extraprotoplasmic matrix (epm) is homogeneous with scattered fibrils and devoid of debris. B) Cross section of the spermatozoid more or less comparable to that of A) showing mitochondrion (m) and unevenly condensed nucleus (n) under a spline (sp). Due to the disrupted development in the mutant, the numerous profiles of flagella (f) are haphazardly arranged, and the cytoplasm debris (cd) fills the extraprotoplasmic matrix (epm). C) Cross section of posterior cell profile showing two flagella (f) and a spline (sp) of +/- 12 microtubules over condensed nucleus (n). The homogeneous extraprotoplasmic matrix (epm) is devoid of cytoplasmic debris and the cytoplasmic remnants (cr) are attached to the cell. D) The mutant has multiple disruptions of the flagella (f), nuclear condensation (n), and spline arrangement (sp). The cytoplasm contains massive and irregular cytoplasmic remnants (cr) and the extraprotoplasmic matrix (epm) contains irregular fibrils and cytoplasmic debris. Bars: A-D = 0.2 μm.


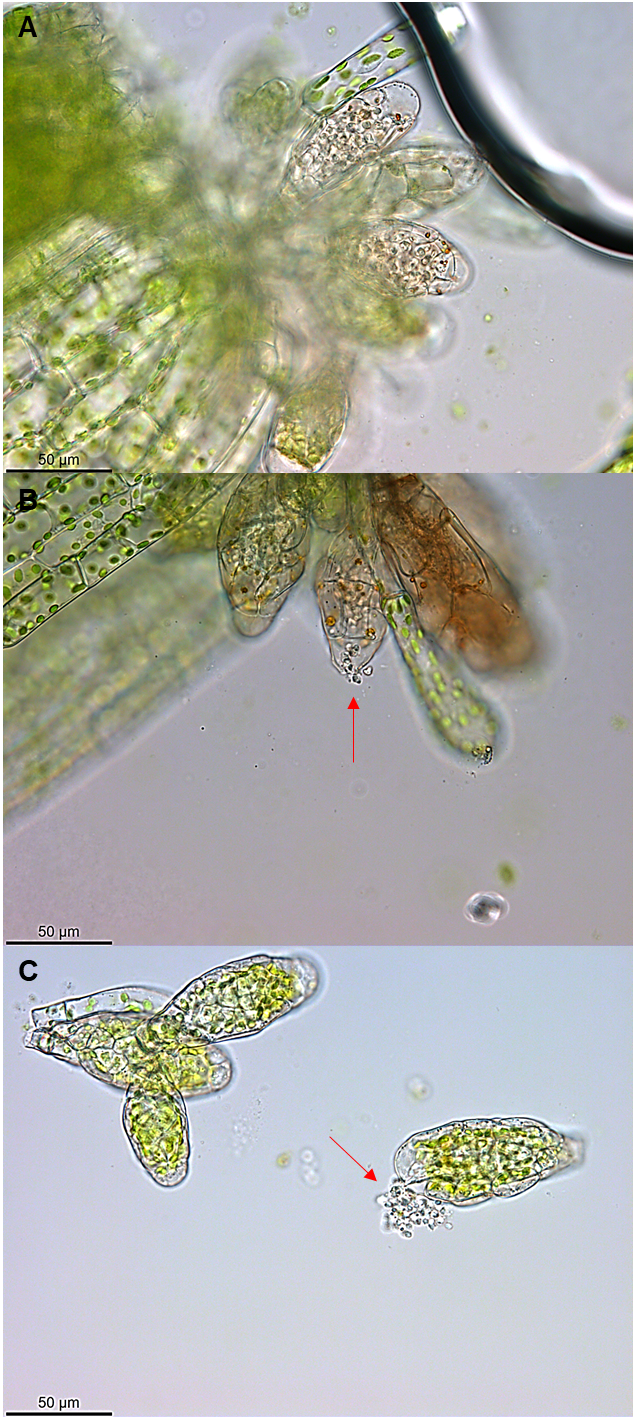


**Figure S15: Pp*hag1* antheridia development 28 dpi.**

A) Closed antheridia turned brownish and discolored. B/C) Opened antheridia released round, bulky spermatozoid agglomerates (indicated by red arrows).


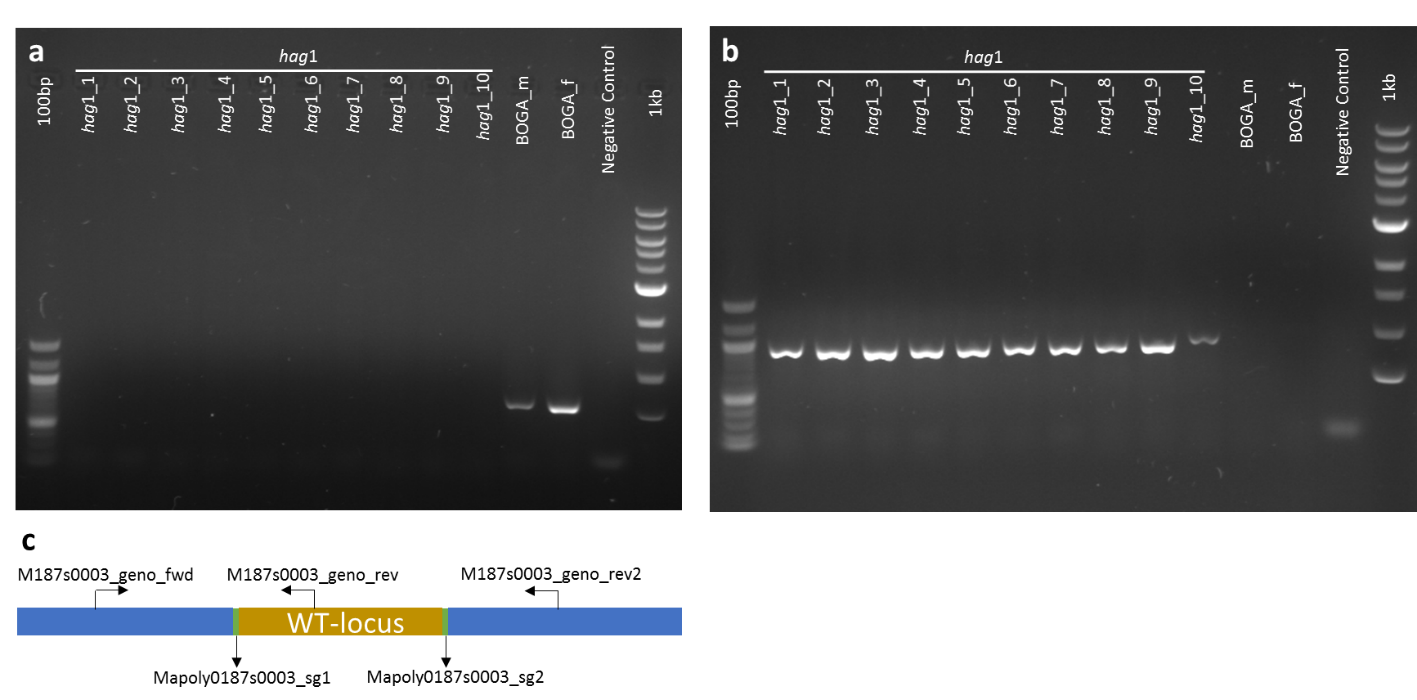


**Figure S16: Genotyping of *M. polymorpha hag1* mutants.**

Presence of the wild type locus was tested using M187s0003_geno_fwd/M187s0003_geno_rev (Table S4). Loss of the wild type locus was verified using M187s0003_geno_fwd/ M187s0003_geno_rev2.


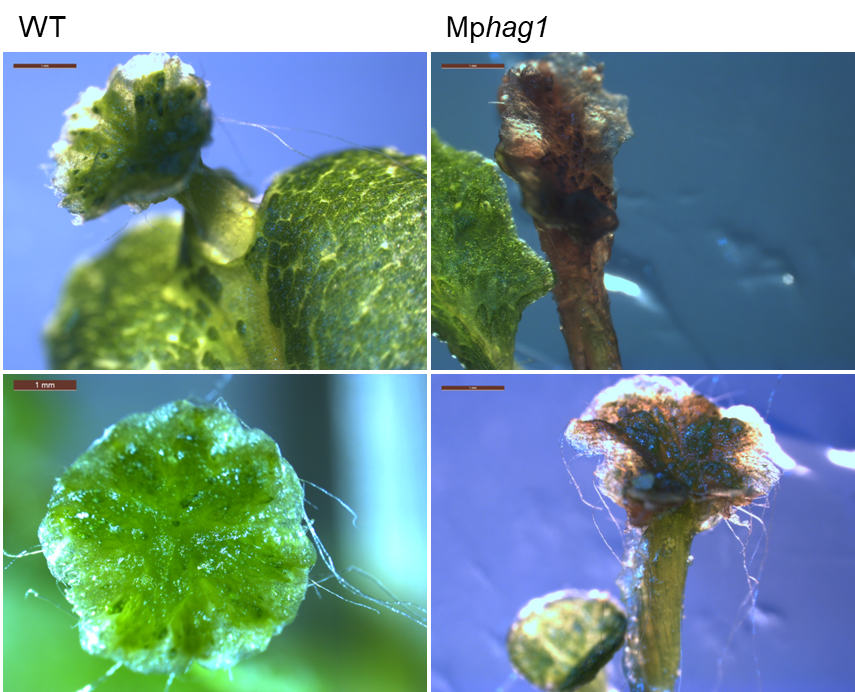


**Figure S17: Antheridiophore development of Mp*hag1* mutants compared to the control.**

Gametangiophores (antheridiophores) developed on top of the thallus after cold and far-red light induction for four weeks. The mutant shows compared to the wild type distorted antheridiophores, occuring before the antheridial receptacle shallowly divided into eight lobes, which has been recognized as a characteristic of antheridial maturity [8].


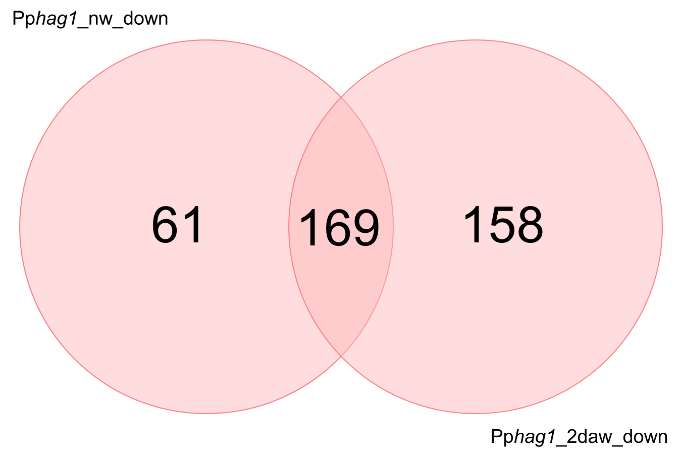

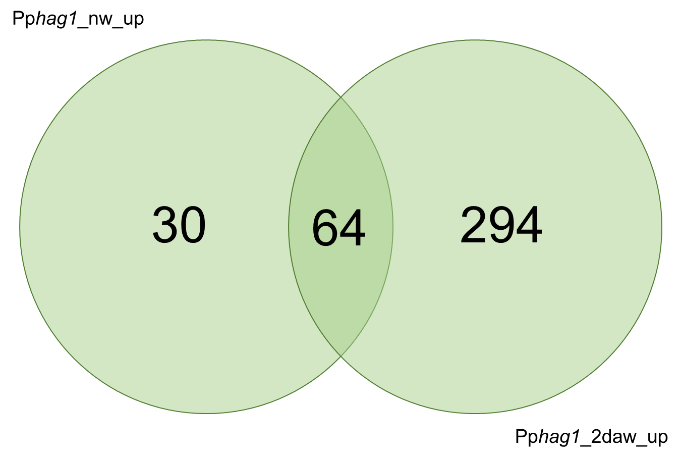

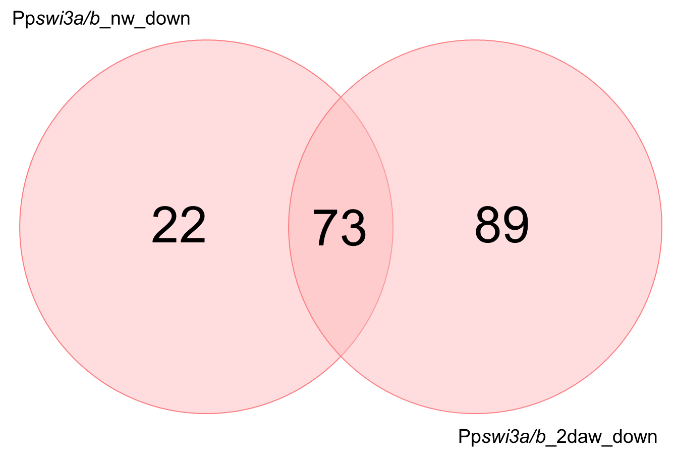

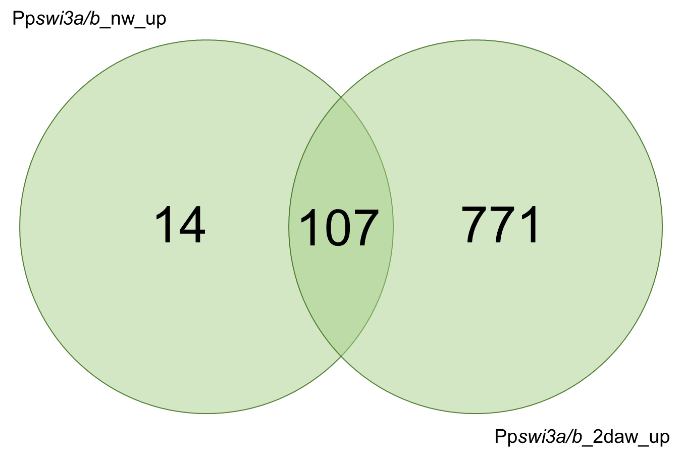


**Figure S18:** **RNA-seq analysis, DEGs.**

Number of DEGs 21 dpi of gametangiogenesis induction (nw = not watered), respectively two days after watering (2daw/23dpi). Each Venn diagram compares the DEGs between the two conditions 21 dpi and 2 daw. The color code indicates up-regulated DEGs (green) in the respective mutant (Pp*hag1*/Pp*swi3a/b*) compared to the control as well as down-regulated DEGs (red) in the respective mutant (Pp*hag1*/Pp*swi3a/b*) compared to the control.


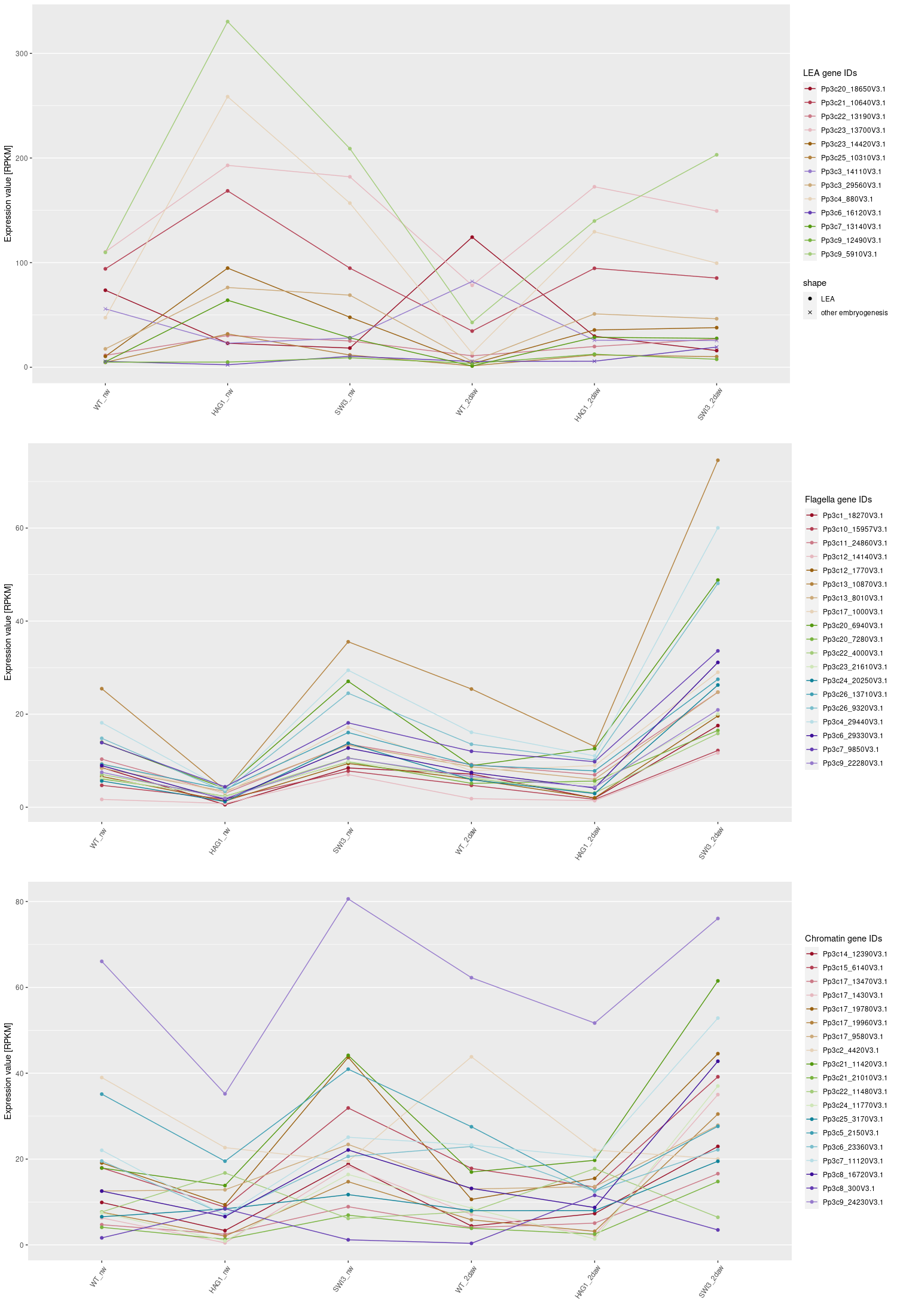


**Figure S19: Expression profile of LEA, flagella and chromatin associated DEGs.**

Genes carrying annotation terms “embryogenesis”, “flagella” or “chromatin” (Table S7) are shown. The expression [RPKM value] of each experiment is shown as the average of RNA-seq sample triplicates. Over all, the DEGs follow a uniform pattern. Embryogenesis/LEA DEGs show higher expression in the HAG1 mutants while flagella and chromatin related DEGs show higher expression in SWI3 mutants. See Results for details.

**Tables**

**Table S1: Candidate gene selection analysed in terms of single copy status via Bayesian inference.**

The number of *P. patens* orthologs is depicted by the arrow colour (green=one, orange=two, red=no). The table lists information of the respective gene, e.g. *P. patens*, *A. thaliana* identifier, TAPscan family, TAIR description/GO biological process [9].

| **TAP family** | ***P. patens* Identifier** | **Bayesian**  **inference** | **TAIR10_id** | **Tair description/GO biological process** |
| --- | --- | --- | --- | --- |
| C2H2 | Pp3c11_480V3.1 | (↑) two  paralogs | AT1G72050 | Encodes a transcriptional factor TFIIIA required for transcription of 5S rRNA gene. 5S rRNA is the smallest constituent of the ribosome. Work on one of the gene models AT1G72050.2 showed that it encodes a protein with nine Cys(2)-His(2)-type zinc fingers, a characteristic feature of TFIIIA proteins. AT1G72050.2 also contains a 23 amino acid spacer between fingers 1 and 2, a 66 amino acid spacer between fingers 4 and 5, and a 50 amino acid non-finger C-terminal tail. in vitro assay demonstrated that AT1g72050.2 binds to 5S rDNA and efficiently stimulates the transcription of 5S rRNA. AT1g72050.2 also binds to 5S rRNA in vitro. AT1g72050.2 is located at several nuclear foci including the nucleolus and is absent from the cytoplasm. multicellular organism development, regulation of transcription, DNA-templated, transcription, DNA-templated |
| FHA | Ppc12_14900V3.1 | ↑ | AT5G47790 | SMAD/FHA domain-containing protein ; CONTAINS InterPro DOMAIN/s: SMAD/FHA domain (InterPro:IPR008984), Forkhead-associated (FHA) domain (InterPro:IPR000253); BEST Arabidopsis thaliana protein match is: SMAD/FHA domain-containing protein (TAIR:AT5G38840.1) |
| GNAT | Pp3c5_21790V3.1 | ↓ no clear  ortholog | AT2G39020 | ornithine metabolic process; Although this locus shares considerable sequence similarity with the adjacent NATA1 gene (At2g39030), they appear to encode genes with different functions. NATA1 is involved in the production of N-delta-acetylornithine, but, overexpression of At2g39020 in tobacco does not lead to the formation of this defense compound. The mRNA is cell-to-cell mobile. |
| GNAT | Pp3c7_5970V3.1 | (↑) two  paralogs | AT5G13780 | embryo development ending in seed dormancy, response to water deprivation |
| GNAT | Pp3c2_9560V3.1 | ↑ | AT3G54610 | Encodes a histone acetyltransferase that is plays a role in the determination of the embryonic root-shoot axis. It is also required to regulate the floral meristem activity by modulating the extent of expression of WUS and AG. In other eukaryotes, this protein is recruited to specific promoters by DNA binding transcription factors and is thought to promote transcription by acetylating the N-terminal tail of histone H3. The enzyme has indeed been shown to catalyse primarily the acetylation of H3 histone with only traces of H4 and H2A/B being acetylated. Non-acetylated H3 peptide or an H3 peptide that had been previously acetylated on K9 both serve as excellent substrates for HAG1-catalyzed acetylation. However, prior acetylation of H3 lysine 14 blocks radioactive acetylation of the peptide by HAG1. HAG1 is specific for histone H3 lysine 14. flower development, histone acetylation, positive regulation of transcription, DNA-templated, regulation of vegetative phase change, response to light stimulus, root morphogenesis, transcription, DNA-templated |
| Jumonji_Other | Pp3c23_15970V3.1 | ↑ | AT5G06550 | Encodes a HR demethylase that acts as a positive regulator of seed germination in the PHYB-PIL5-SOM pathway. cell surface receptor signaling pathway, histone H4-R3 methylation, positive regulation of seed germination |
| Med7 | Pp3c5_20850V3.1 | (↑) two  paralogs | AT5G03500 | Mediator complex, subunit Med7; FUNCTIONS IN: RNA polymerase II transcription mediator activity; INVOLVED IN: regulation of transcription from RNA polymerase II promoter; LOCATED IN: mediator complex; CONTAINS InterPro DOMAIN/s: Mediator complex, subunit Med7 (InterPro:IPR009244); BEST Arabidopsis thaliana protein match is: Mediator complex, subunit Med7 (TAIR:AT5G03220.1) |
| MYB-related | Pp3c2_24980V3.1 | ↑ | AT2G47620 | chromatin remodelling, covalent chromatin modification, multicellular organism development, regulation of transcription, DNA-templated, transcription, DNA-templated |
| MYB-related | Pp3c14_5130V3.1 | (↑) two  paralogs | AT5G06110 | DnaJ domain ;Myb-like DNA-binding domain; FUNCTIONS IN: heat shock protein binding, DNA binding; INVOLVED IN: protein folding; EXPRESSED IN: 23 plant structures; EXPRESSED DURING: 13 growth stages; CONTAINS InterPro DOMAIN/s: Molecular chaperone, heat shock protein, Hsp40, DnaJ (InterPro:IPR015609), Heat shock protein DnaJ, N-terminal (InterPro:IPR001623), Heat shock protein DnaJ, conserved site (InterPro:IPR018253), MYB-like (InterPro:IPR017877), SANT, DNA-binding (InterPro:IPR001005), Myb, DNA-binding (InterPro:IPR014778), Homeodomain-like (InterPro:IPR009057); BEST Arabidopsis thaliana protein match is: DnaJ domain ;Myb-like DNA-binding domain (TAIR:AT3G11450.1) cell division, protein folding |
| SET/Sigma70-like | Pp3c14_4440V3.1 | ↑ | AT1G64860 | DNA-templated transcription, initiation, cellular response to light stimulus, cellular response to redox state, photosystem stoichiometry adjustment, regulation of RNA biosynthetic process, regulation of transcription, DNA-templated |
| TUB | Pp3c3_17070V3.1 | ↓ no clear  ortholog | AT2G47900 | cellular response to osmotic stress, regulation of transcription, DNA-templated, response to fungus, response to hydrogen peroxide, response to salt stress |

**Table S2: Detailed numbers of crossing analyses with a fluorescent male fertile strain to test for male impairment.**

Pp*swi3a/b* and Pp*hag1* were crossed with Re-mcherry according to [10]. Shown is the number of sporophytes per gametophore (s/g) in percentage relative to the total number of gametophores, and the rate of crosses per sporophytes (c/s) in percentage relative to the total number of sporophytes. Most of the sporophytes in the Reute control derive from selfing (homozygous; hence low number of heterozygous sporophytes, c/s). In contrast, almost 100% of mutant sporophytes are heterozygous, indicating a male impairment. The cross with the male fertile strain could largely restore the phenotype in *swi3a/b* (sporophyte/gametophore ratio at least 80%), while Pp*hag1* shows a significantly reduced sporophyte ratio as compared to the control. Three independent replicates were performed for the three mutant lines as well as the control. The total number of gametophores analysed was 621 (*swi3a/b*), 770 (*hag1*) and 190 (control).

| **Mutant/control** | **replicate 1 (%)** | **replicate 2 (%)** | **replicate 3 (%)** |
| --- | --- | --- | --- |
| **swi3a/b_1 s/g** | 100.00 | 82.26 | 98.81 |
| **swi3a/b_1 cs** | 96.05 | 100.00 | 98.80 |
| **swi3a/b_2 s/g** | 100.00 | 79.78 | 87.04 |
| **swi3a/b_2 cs** | 100.00 | 97.18 | 100.00 |
| **swi3a/b_3 s/g** | 97.33 | 86.59 | 100.00 |
| **swi3a/b_3 cs** | 100.00 | 94.37 | 96.49 |
| **hag1_1 s/g** | 21.21 | 14.08 | 4.00 |
| **hag1_1 cs** | 100.00 | 100.00 | 100.00 |
| **hag1_2 s/g** | 60.61 | 32.89 | 57.14 |
| **hag1_2 cs** | 100.00 | 96.00 | 100.00 |
| **hag1_3 s/g** | 28.81 | 22.09 | 46.35 |
| **hag1_3 cs** | 82.35 | 100.00 | 96.63 |
| **Reute s/g** | 95.45 | 95.83 | 100.00 |
| **Reute cs** | 3.17 | 2.90 | 3.85 |

**Table S3: see extra file describing the sources of the genome-derived protein sets used.**

**Table S4: List of PCR primers used in this study.**

| **name** | **sequence (5' 🡪 3')** |
| --- | --- |
| Mapoly0187s0003_sg1_rev | AAACAGTGGATTGTGGCTCTACCC |
| Mapoly0187s0003_sg2_rev | AAACCCGCAAACATGTCCAGCGTC |
| M13for | GTAAAACGACGGCCAGT |
| M13rev | GGAAACAGCTATGACCATG |
| Mapoly0187s0003#1 | ACGTACATGAGAGTACGAAAGCA |
| Mapoly0187s0003#2 | ATACTCCTTTGTTGCACAGATGC |
| Mapoly0187s0003#3 | TCACGACTGCAGTACGCTC |
| Mapoly0187s0003_sg1_for | CTCGGGGTAGAGCCACAATCCACT |
| Mapoly0187s0003_sg2_for | CTCGGACGCTGGACATGTTTGCGG |
| Marpo_female_fwd | CACCATGGGCCTACTTGTTTCAGTCGCTGGTGG |
| Marpo_female_rev | TCAAAGGCTAGTGTTTCCATTACTTGGAC |
| Marpo_male_fwd | GCAGCTGTGTTTTGTGCAGATCGTC |
| Marpo_male_rev | ATTCTGACCTTACAAGAAATCCTCC |
| pJet_rev | GCTGAGAATATTGTAGGAGATCTTCTAG |
| pJet_uni_neu | CAACTGCTTTAACACTTGTGCCTG |
| Pp3c2_24980#1 | TGCTTCCAAGGAGGCGAAAC |
| Pp3c2_24980#2 | ACTATGCAACTCCATCACTCCAGG |
| Pp3c2_24980#3-EcoR1 | GAATTCATGGTGAACCCGGCG |
| Pp3c2_24980#4-Xba1 | TCTAGATGAACTGGGCGCAGGG |
| Pp3c2_9560#1 | TCCAACGGTGATTCTCGCAG |
| Pp3c2_9560#2 | AGGTGCTCCCGAGATACG |
| Pp3c2_9560#3 | TCGTCAGGATCCGTTCCAATG |
| Pp3c2_9560#4-EcoR1 | GAATTCATGCGCAAGGGGGCC |
| Pp3c2_9560#5-Sal1 | GTCGACTGACAAGTGTTGTGATCTGCTGACCAAC |

**Table S5: List of sgRNAs expression cassettes used in this study.**

Promoter sequences are in blue, target sequences in red and tracrRNA sequences in green. For *M. polymorpha* tracerRNA and promoter were already implemented in the vectors ordered from Addgene.

| Name | Sequence (5'-3') |
| --- | --- |
| Pp3c2_9560-sgRNA1 | GTCCATTGAAGCAGACGTGTTGCGACAGGTTAGCGACGATGGGTGTAGATGTGATGTGATGTGATGGTGTGGTTCTTCCACGGCGGCGTCCTTGCGGTGGCGGAGAAGGGGATATCCCGAAGGAGCGGCAGCGGGAGAGCACAAGCAGAAAGGGTGCAGTGAGTGAGTGGGTCCAGCTGGGTGGCTGGCCGAGTGGACGCGACCGGGTTTCGAGGGGGGGGGGGAGAAAAGGGATGGAGCGAGGGATATAACCCACATGGAATGGAGGTGGGTGTGAAGGCGGGTATATAGGAAGGTGGAGGACTTACAACCCATgccaccaatcttctcaacggGTTTTAGAGCTAGAAATAGCAAGTTAAAATAAGGCTAGTCCGTTATCAACTTGAAAAAGTGGCACCGAGTCGGTGCTTTTTTT |
| Pp3c2_9560-sgRNA2 | GTCCATTGAAGCAGACGTGTTGCGACAGGTTAGCGACGATGGGTGTAGATGTGATGTGATGTGATGGTGTGGTTCTTCCACGGCGGCGTCCTTGCGGTGGCGGAGAAGGGGATATCCCGAAGGAGCGGCAGCGGGAGAGCACAAGCAGAAAGGGTGCAGTGAGTGAGTGGGTCCAGCTGGGTGGCTGGCCGAGTGGACGCGACCGGGTTTCGAGGGGGGGGGGGAGAAAAGGGATGGAGCGAGGGATATAACCCACATGGAATGGAGGTGGGTGTGAAGGCGGGTATATAGGAAGGTGGAGGACTTACAACCCATggttcgaaggtgcgaacggGTTTTAGAGCTAGAAATAGCAAGTTAAAATAAGGCTAGTCCGTTATCAACTTGAAAAAGTGGCACCGAGTCGGTGCTTTTTTT |
| Pp3c2_24980-sgRNA1 | GTCCATTGAAGCAGACGTGTTGCGACAGGTTAGCGACGATGGGTGTAGATGTGATGTGATGTGATGGTGTGGTTCTTCCACGGCGGCGTCCTTGCGGTGGCGGAGAAGGGGATATCCCGAAGGAGCGGCAGCGGGAGAGCACAAGCAGAAAGGGTGCAGTGAGTGAGTGGGTCCAGCTGGGTGGCTGGCCGAGTGGACGCGACCGGGTTTCGAGGGGGGGGGGGAGAAAAGGGATGGAGCGAGGGATATAACCCACATGGAATGGAGGTGGGTGTGAAGGCGGGTATATAGGAAGGTGGAGGACTTACAACCCATgccggagcccactcccgaagGTTTTAGAGCTAGAAATAGCAAGTTAAAATAAGGCTAGTCCGTTATCAACTTGAAAAAGTGGCACCGAGTCGGTGCTTTTTTT |
| Pp3c2_24980-sgRNA2 | GTCCATTGAAGCAGACGTGTTGCGACAGGTTAGCGACGATGGGTGTAGATGTGATGTGATGTGATGGTGTGGTTCTTCCACGGCGGCGTCCTTGCGGTGGCGGAGAAGGGGATATCCCGAAGGAGCGGCAGCGGGAGAGCACAAGCAGAAAGGGTGCAGTGAGTGAGTGGGTCCAGCTGGGTGGCTGGCCGAGTGGACGCGACCGGGTTTCGAGGGGGGGGGGGAGAAAAGGGATGGAGCGAGGGATATAACCCACATGGAATGGAGGTGGGTGTGAAGGCGGGTATATAGGAAGGTGGAGGACTTACAACCCATgcactccagaggctgcgaccGTTTTAGAGCTAGAAATAGCAAGTTAAAATAAGGCTAGTCCGTTATCAACTTGAAAAAGTGGCACCGAGTCGGTGCTTTTTTT |
| Mapoly0187s0003.1_sgRNA1 | CTACTACACTCCTCAACGCAAGTTCTATCTCATATCTCTGCAAGAGCTTATTCCAAGAAACTTTCTTGTGCTTCTATGCATGCCCAGGTACCTTAAGTCGCGCGCATAGTCACTTCACAGCCTTTTCAGTTCTCCCTTTGATGGCTCAGAGTGACGCAACTGCACAAAAATAAGAAAAAAGTCGAAGCGTCACGAGAGATGTATCCGGCTAGTTTCCTTGGAGTTCGGATTGCTCTCTTTCTCTGTCTCCCCGAACCATATGGAATTTATGAACTGCTGTTCACTAGTCAGTCACTTGTTTCAGAACTTTTGAGTGGTCGATTTATCCACCTTCTTGTTTCTTCCTGGGTCCAGAATCTGACTTCTTGAGACAACAAACAGTGCACAGTGGATCGAAGAATGACCACAGTTGAGACACGTGGCGCTGGCCTCATTTATTGCACCATACGTTTAGTGAAGAACTGAATAATGGTGGATATAAAGCAGAGTTGCACCCAGCCTCTgggtagagccacaatccactGTTTTAGAGCTAGAAATAGCAAGTTAAAATAAGGCTAGTCCGTTATCAACTTGAAAAAGTGGCACCGAGTCGGTGCTTTTTTT |
| Mapoly0187s0003.1_sgRNA2 | CTACTACACTCCTCAACGCAAGTTCTATCTCATATCTCTGCAAGAGCTTATTCCAAGAAACTTTCTTGTGCTTCTATGCATGCCCAGGTACCTTAAGTCGCGCGCATAGTCACTTCACAGCCTTTTCAGTTCTCCCTTTGATGGCTCAGAGTGACGCAACTGCACAAAAATAAGAAAAAAGTCGAAGCGTCACGAGAGATGTATCCGGCTAGTTTCCTTGGAGTTCGGATTGCTCTCTTTCTCTGTCTCCCCGAACCATATGGAATTTATGAACTGCTGTTCACTAGTCAGTCACTTGTTTCAGAACTTTTGAGTGGTCGATTTATCCACCTTCTTGTTTCTTCCTGGGTCCAGAATCTGACTTCTTGAGACAACAAACAGTGCACAGTGGATCGAAGAATGACCACAGTTGAGACACGTGGCGCTGGCCTCATTTATTGCACCATACGTTTAGTGAAGAACTGAATAATGGTGGATATAAAGCAGAGTTGCACCCAGCCTCTgacgctggacatgtttgcggGTTTTAGAGCTAGAAATAGCAAGTTAAAATAAGGCTAGTCCGTTATCAACTTGAAAAAGTGGCACCGAGTCGGTGCTTTTTTT |

**Table S6: Detailed numbers of gametangiophore development analyses for *Marchantia polymorpha*.**

Three independent replicates (three ECO2 boxes with two plants respectively) were analysed with regard to the mutant lines as well as the wild type (WT; m=male, f=female). The mutant lines were numbered consecutively.

| **Lines** | **Replicate 1** | | **Replicate 2** | | **Replicate 3** | |
| --- | --- | --- | --- | --- | --- | --- |
|  | Plant 1 | Plant 2 | Plant 1 | Plant 2 | Plant 1 | Plant 2 |
| ***hag1*_1** | 0 | 0 | 4 | 2 | 0 | 0 |
| ***hag1*_2** | 0 | 0 | 0 | 1 | 0 | 0 |
| ***hag1*_3** | 0 | 1 | 0 | 0 | 0 | 0 |
| ***hag1*_4** | 0 | 0 | 0 | 1 | 0 | 0 |
| ***hag1*_5** | 0 | 0 | 0 | 0 | 0 | 0 |
| ***hag1*_6** | 0 | 0 | 6 | 0 | 12 | 2 |
| ***hag1*_7** | 0 | 0 | 0 | 1 | 0 | 0 |
| ***hag1*_8** | 4 | 3 | 25 | 4 | 0 | 0 |
| ***hag1*_9** | 2 | 1 | 0 | 1 | 0 | 1 |
| ***hag1*_10** | 0 | 0 | 1 | 0 | 0 | 0 |
| **WT_m** | 32 | 32 | 20 | 21 | 49 | 40 |
| **WT_f** | 40 | 38 | 38 | 50 | 43 | 34 |

**Table S7: DEGs annotated as related to embryogenesis (blue; yellow: LEA), chromatin (orange) and flagella (green).**

**References**

1. Lechner M, Findeiss S, Steiner L, Marz M, Stadler PF, Prohaska SJ: **Proteinortho: detection of (co-)orthologs in large-scale analysis**. *BMC Bioinformatics* 2011, **12**:124.

2. Wilhelmsson PKI, Muhlich C, Ullrich KK, Rensing SA: **Comprehensive Genome-Wide Classification Reveals That Many Plant-Specific Transcription Factors Evolved in Streptophyte Algae**. *Genome Biol Evol* 2017, **9**(12):3384-3397.

3. Frickenhaus S, Beszteri B: **Quicktree-SD, Software developed by AWI-Bioinformatics**. In*.*; 2008.

4. Fernandez-Pozo N, Haas FB, Meyberg R, Ullrich KK, Hiss M, Perroud PF, Hanke S, Kratz V, Powell AF, Vesty EF *et al*: **PEATmoss (Physcomitrella Expression Atlas Tool): a unified gene expression atlas for the model plant Physcomitrella patens**. *Plant J* 2019.

5. Meyberg R, Perroud PF, Haas FB, Schneider L, Heimerl T, Renzaglia KS, Rensing SA: **Characterization of evolutionarily conserved key players affecting eukaryotic flagellar motility and fertility using a moss model**. *New Phytol* 2020.

6. Ortiz-Ramirez C, Hernandez-Coronado M, Thamm A, Catarino B, Wang M, Dolan L, Feijo JA, Becker JD: **A transcriptome atlas of *Physcomitrella patens* provides insights into the evolution and development of land plants**. *Mol Plant* 2015, **9**:205-220.

7. Perroud PF, Haas FB, Hiss M, Ullrich KK, Alboresi A, Amirebrahimi M, Barry K, Bassi R, Bonhomme S, Chen H *et al*: **The Physcomitrella patens gene atlas project: large-scale RNA-seq based expression data**. *Plant J* 2018, **95**(1):168-182.

8. Shimamura M: **Marchantia polymorpha : Taxonomy, Phylogeny and Morphology of a Model System**. *Plant and Cell Physiology* 2015, **57**(2):230-256.

9. Lang D, Ullrich KK, Murat F, Fuchs J, Jenkins J, Haas FB, Piednoel M, Gundlach H, Van Bel M, Meyberg R *et al*: **The Physcomitrella patens chromosome-scale assembly reveals moss genome structure and evolution**. *Plant J* 2018, **93**(3):515-533.

10. Perroud PF, Meyberg R, Rensing SA: **Physcomitrella patens Reute mCherry as a tool for efficient crossing within and between ecotypes**. *Plant Biol (Stuttg)* 2019, **21 Suppl 1**:143-149.
